# Supplementary material for: Mapping person-to-person variation in viral mutations that escape polyclonal serum targeting influenza hemagglutinin
Source: eLife. 2019 Aug 27;8:e49324. doi: 10.7554/eLife.49324 (PMC6711711; doi:10.7554/eLife.49324)
Supplement: Supplementary file 5. [file elife-49324-supp5.zip › supplementary_file_5.html]

analyze\_revgen


 


# Analysis of Perth/2009 H3 HA mutant libraries generated by reverse genetics¶

We generated mutant virus libraries by reverse genetics to use for mutational antigenic profiling of serum.
In the following notebook, we will analyze the deep sequencing data of the mutant libraries of the Perth/2009 H3 HA that we generated using reverse genetics.
This includes analysis of the mutant plasmid libraries created in the pHH21 backbone (Neumann et al (1999)), and the mutant virus libraries generated by reverse genetics and passaged in MDCK-SIAT1-TMPRSS2 cells (Lee et al (2018)) to select for functional HA variants.

## Experimental overview¶

In brief, we used two rounds of a PCR-based approach, described in Bloom (2014) and Dingens et al (2017) to create codon mutant libraries.
We created these libraries in biological triplicate in the background of the Perth/2009 H3 HA carrying two mutations, G78D and T212I.
As described in Lee et al (2018), these two mutations were introduced as they improved viral growth in cell culture.

Next we used the mutant plasmid library to generate the initial pool of mutant virus libraries by reverse genetics.
This approach involved transfecting a co-culture of 293T and MDCK-SIAT1-TMPRSS2 cells with equal amounts of the Perth/2009 HA mutant plasmid library, the seven `pHW*` series of plasmids encoding each of the WSN/1933 internal genes and the WSN/1933 NA, and the pHAGE2-EF1aInt-TMPRSS2-IRES-mCherry-W protein expression plasmid Lee et al (2018).
We then passaged the rescued mutant libraries at low multiplicity of infection in MDCK-SIAT1-TMPRSS2 cells to select for viruses carrying functional HA variants.

For each library, there is a *mutDNA-#* sample which represents the plasmid mutant library, and a *mutvirus-#* sample which represents the mutant viruses *after* passaging.
There are also a set of wild-type controls for the unmutated plasmid and the virus, referred to as *wtDNA* and *wtvirus*.

As described previously, all of the plasmid and virus samples were deep sequenced using a barcoded-subamplicon sequencing approach to sequence the samples to high accuracy.

## Import modules, define directories¶

In [1]:

```
import os
import re
import glob
import subprocess
import pandas as pd
import seaborn as sns
import collections

import dms_tools2
import dms_tools2.sra
import dms_tools2.plot
import dms_tools2.prefs
from dms_tools2.ipython_utils import showPDF
import warnings
warnings.simplefilter('ignore') # don't print warnings to avoid clutter in notebook
from IPython.display import display, HTML, Markdown
# from pymodules.utils import * # custom modules for this analysis

print("Using dms_tools2 version {0}".format(dms_tools2.__version__))

# CPUs to use, should not exceed the number you request with slurm
ncpus = -1

# do we use existing results or generate everything new?
use_existing = 'yes'

inputdir = './data/'

resultsdir = './results/'
fastqdir = os.path.join(resultsdir, 'FASTQ_files/')
countsdir = os.path.join(resultsdir, 'codoncounts/')
plotsdir = os.path.join(resultsdir, 'plots/')

for d in [resultsdir, fastqdir, countsdir, plotsdir]:
    os.makedirs(d, exist_ok=True)
```

```
Using dms_tools2 version 2.4.12
```

## Display Sanger sequencing data¶

We first Sanger sequenced 29 random clones from the mutant plasmid libraries to get an estimate of the mutation rate and mutation composition.
The results of this analysis are shown in the plots below.
The plots can be found in the subdirectory data/SangerAnalysis/.

The average number of substitutions for the mutant plasmid library is **1.6 codon substitutions** per clone.

In [2]:

```
sangerprefix = './data/SangerAnalysis/'
```

In [3]:

```
showPDF(sangerprefix + 'codonmutntcomposition.pdf')
```

The plot above shows that the nucleotide frequencies in the codon mutations were relatively uniform, with perhaps a slightly reduced frequency of `A` relative to the other three nucleotides.

In [4]:

```
showPDF(sangerprefix + 'mutationclustering.pdf')
```

The distance between pairs of mutations is also relatively as expected up to 200 nucleotides, with perhaps some more mutation pairs being farther than 200 nt apart than expected.

In [5]:

```
showPDF(sangerprefix + 'mutpositions.pdf')
```

The plot above shows the positions of the codon substitutions and any indels for each of the 29 clones. Most clones have single codon mutations, while a few have double and one has triple.

In [6]:

```
showPDF(sangerprefix + 'mutpositions_cumulative.pdf')
```

From the plot above, the mutations appear to be relatively evenly distributed across the entire HA gene and mutations are not particularly enriched in a specific region of the gene.

In [7]:

```
showPDF(sangerprefix + 'ncodonmuts.pdf')
```

It appears that single, double, and triple-nucleotide mutations are all present.
Single-nucleotide mutations are slighly enriched relative to what is expected, while double mutations are slightly depleted compared to expected.

In [8]:

```
showPDF(sangerprefix + 'nmutdist.pdf')
```

The Sanger sequencing analysis showed an average of 1.6 codon substitutions per clone, which is slightly higher than that of the mutant plasmid library used in Lee et al (2018).
The distribution of codon mutations per clone does not strictly follow a Poisson distribution, but there are many single mutants present, with a mixture of clones with 2 or more codon mutations.

## Define the samples¶

Here we will create a `pandas` dataframe to define the samples with the following information:

1. The **name** of each sample.
2. The SRA run accession for the deep sequencing data.

In [9]:

```
samples = pd.DataFrame.from_records(
        [('mutDNA-1', 'SRR9201667'),
         ('mutDNA-2', 'SRR9201664'),
         ('mutDNA-3', 'SRR9201663'),
         ('mutvirus-1', 'SRR9201668'),
         ('mutvirus-2', 'SRR9201672'),
         ('mutvirus-3', 'SRR9201671'),
         ('wtDNA', 'SRR9201666'),
         ('wtvirus', 'SRR9201669')],
        columns=['name', 'run']
        )
```

## Download `FASTQ` files from the SRA¶

All of the FASTQ files are on the Sequence Read Archive (SRA) under the run numbers listed in the `samples` dataframe defined above. To download these files, we just pass that dataframe to the dms\_tools2.sra.fastqFromSRA function. Note that this requires the `fastq-dump` and `aspera` programs to be installed on the computer you are using at the specified paths.

In [10]:

```
print('Downloading FASTQ files from the SRA...')
dms_tools2.sra.fastqFromSRA(
        samples=samples,
        fastq_dump='fastq-dump', # valid path to this program on the Hutch server
        fastqdir=fastqdir,
        aspera=(
            '/app/aspera-connect/3.7.5/bin/ascp', # valid path to ascp on Hutch server
            '/app/aspera-connect/3.7.5/etc/asperaweb_id_dsa.openssh' # Aspera key on Hutch server
            ),
        overwrite={'no':True, 'yes':False}[use_existing],
        )
print('Completed download of FASTQ files from the SRA')

print('Here are the names of the downloaded files now found in {0}'.format(fastqdir))
display(HTML(samples.to_html(index=False)))
```

```
Downloading FASTQ files from the SRA...
Completed download of FASTQ files from the SRA
Here are the names of the downloaded files now found in ./results/FASTQ_files/
```

| name | run | R1 | R2 |
| --- | --- | --- | --- |
| mutDNA-1 | SRR9201667 | mutDNA-1\_R1.fastq.gz | mutDNA-1\_R2.fastq.gz |
| mutDNA-2 | SRR9201664 | mutDNA-2\_R1.fastq.gz | mutDNA-2\_R2.fastq.gz |
| mutDNA-3 | SRR9201663 | mutDNA-3\_R1.fastq.gz | mutDNA-3\_R2.fastq.gz |
| mutvirus-1 | SRR9201668 | mutvirus-1\_R1.fastq.gz | mutvirus-1\_R2.fastq.gz |
| mutvirus-2 | SRR9201672 | mutvirus-2\_R1.fastq.gz | mutvirus-2\_R2.fastq.gz |
| mutvirus-3 | SRR9201671 | mutvirus-3\_R1.fastq.gz | mutvirus-3\_R2.fastq.gz |
| wtDNA | SRR9201666 | wtDNA\_R1.fastq.gz | wtDNA\_R2.fastq.gz |
| wtvirus | SRR9201669 | wtvirus\_R1.fastq.gz | wtvirus\_R2.fastq.gz |

## Align deep sequencing data and count mutations¶

We have employed a barcoded-subamplicon sequencing approach to deep sequence the samples to high accuracy. We will now analyze the sequence data using the `dms2_batch_bcsubamp` program.

In [11]:

```
# Reference coding sequence of Perth/2009 HA; from plasmid #1535 pICR2-Perth09-HA
refseq = './data/Perth09_HA_reference.fa'

# Temporary align specs
alignspecs = ' '.join(['1,285,38,40', '286,567,33,34', '568,852,34,30', 
                       '853,1137,34,31', '1138,1422,36,29', '1423,1701,39,44'])

# write sample information to a batch file for dms2_batch_bcsubamplicons
countsbatchfile = os.path.join(countsdir, 'batch.csv')
samples.to_csv(countsbatchfile, index=False)

print('\nNow running dms2_batch_bcsubamp...')
log = !dms2_batch_bcsubamp \
        --batchfile {countsbatchfile} \
        --refseq {refseq} \
        --alignspecs {alignspecs} \
        --outdir {countsdir} \
        --summaryprefix summary \
        --R1trim 200 --R2trim 165 \
        --fastqdir {fastqdir} \
        --ncpus {ncpus} \
        --use_existing {use_existing}

print('Completed dms2_batch_bcsubamp.')
```

```
Now running dms2_batch_bcsubamp...
Completed dms2_batch_bcsubamp.
```

In [12]:

```
!ls {countsdir}/*_codoncounts.csv
```

```
./results/codoncounts//mutDNA-1_codoncounts.csv
./results/codoncounts//mutDNA-2_codoncounts.csv
./results/codoncounts//mutDNA-3_codoncounts.csv
./results/codoncounts//mutvirus-1_codoncounts.csv
./results/codoncounts//mutvirus-2_codoncounts.csv
./results/codoncounts//mutvirus-3_codoncounts.csv
./results/codoncounts//wtDNA_codoncounts.csv
./results/codoncounts//wtvirus_codoncounts.csv
```

In [13]:

```
countsplotprefix = os.path.join(countsdir, 'summary')
```

In [14]:

```
showPDF(countsplotprefix + '_readstats.pdf', width=500)
```

In [15]:

```
showPDF(countsplotprefix + '_readsperbc.pdf')
```

From the reads per barcode plot above, it appears that most of the samples have good sequencing depth, although mutDNA-1 looks a bit undersequenced.

In [16]:

```
showPDF(countsplotprefix + '_bcstats.pdf', width=500)
```

The plot above shows that the majority of reads are aligned.

In [17]:

```
showPDF(countsplotprefix + '_depth.pdf')
```

The sequencing depth across the HA gene appears to be fairly even for each sample. There are small dips near the middle of the subamplicon where sequencing quality drops near the end of R1 and R2. However, we've minimized the number of reads thrown out by poor sequencing quality by trimming each read.

In [18]:

```
showPDF(countsplotprefix + '_mutfreq.pdf')
```

The mutation frequencies are highest in the mutDNA samples and lowest in the wtDNA and wtvirus samples. There is a spike in mutation frequency at the first codon in each of the three mutDNA samples as well as the mutvirus samples. This is similar to the observation of a spike in mutation frequency at the first codon for the plasmid mutant libraries in Lee et al (2018).
There are also spikes in the mutation rate near the end of the HA gene for the mutant plasmid libraries, which may be due to the relatively high mutational tolerance of this region, also described in Lee et al (2018).

In [19]:

```
showPDF(countsplotprefix + '_cumulmutcounts.pdf')
```

The cumulative mutation counts plot above shows that nearly all codon and amino-acid mutations were sampled at least once in the mutant DNA samples, as desired.
The number of mutations with at least one count decreases for each of the mutant virus libraries relative to the DNA libraries, suggesting that mutations are lost upon rescue or passaging.

In [20]:

```
showPDF(countsplotprefix + '_codonmuttypes.pdf', width=500)
```

The mutation frequencies of the wildtype plasmid and the wildtype virus controls are low as expected, and the wildtype virus has a slightly higher mutation frequency than the wildtype plasmid does, because viral replication is an error-prone process. These mutations are exclusively nonsynonymous and synonymous mutations.
After error correction, the frequencies of the stop and nonsynonymous mutations in the passaged mutant viruses have dropped relative to their starting frequencies in the mutant plasmid libraries, strongly suggesting that we are selecting against presumably non-functional HA variants.
However, there is some incomplete purging of stop codons, which was also observed after passaging of the helper-virus libraries.

In [21]:

```
showPDF(countsplotprefix + '_codonntchanges.pdf', width=500)
```

All of the mutations in the wtvirus are single nucleotide mutations, which is as expected.
The mutDNA libraries show many single, double, and triple nucleotide mutations.
The overall mutation frequency in the mutant viruses is reduced, but 1, 2, and 3 nucleotide mutations are all present.

In [22]:

```
showPDF(countsplotprefix + '_singlentchanges.pdf', width=500)
```

Although there might be a bit of oxidative damage (`C to A` and `G to T` mutations) in the mutDNA, this doesn't appear to be substantial and we see a fairly even distribution of nucleotide changes, with some enrichment for transition mutations.

In [ ]:

```

```
