# Supplementary figures and images for: Mapping person-to-person variation in viral mutations that escape polyclonal serum targeting influenza hemagglutinin
Source: eLife. 2019 Aug 27;8:e49324. doi: 10.7554/eLife.49324 (PMC6711711; doi:10.7554/eLife.49324)

differential selection = 11

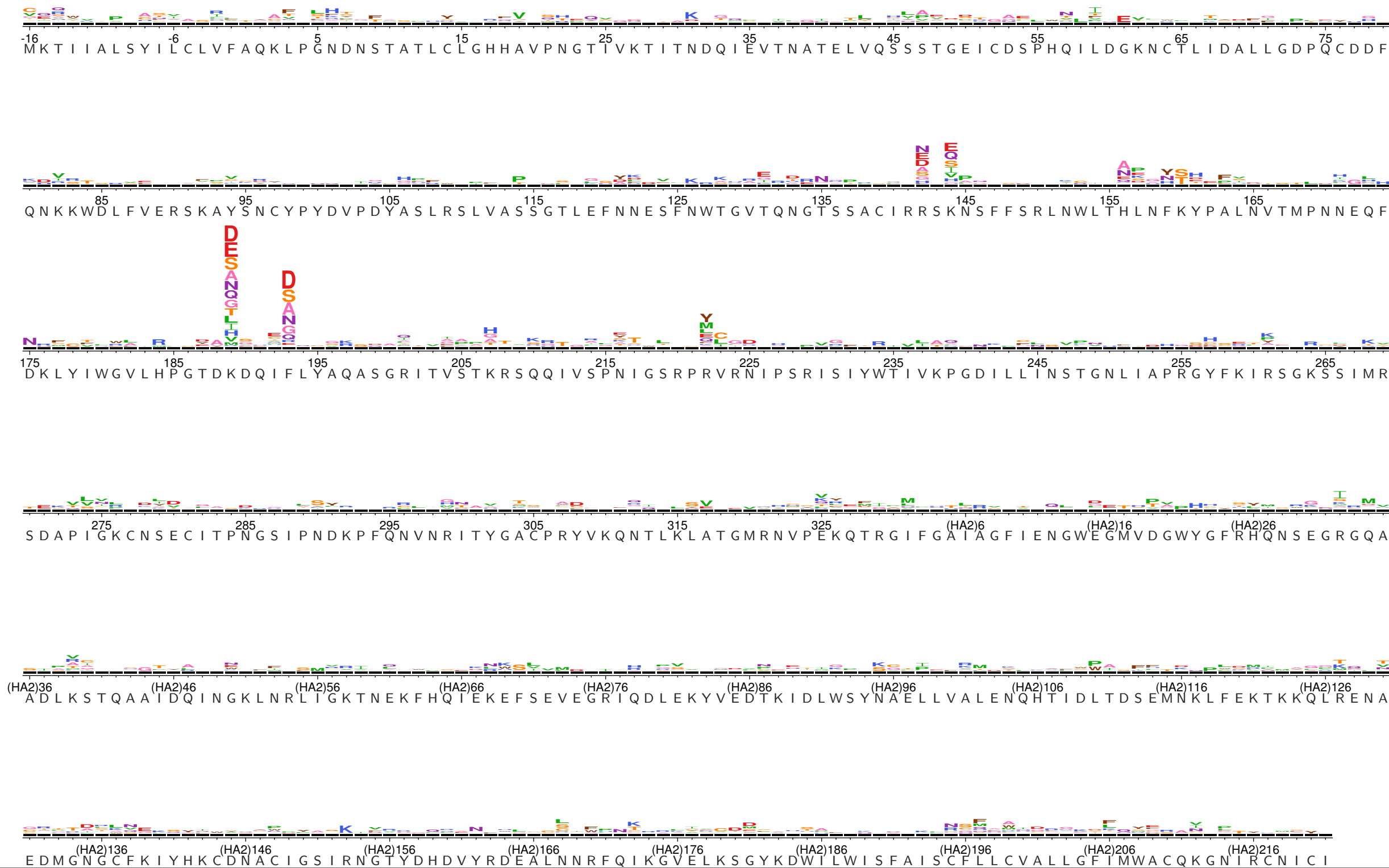

Supplement: Supplementary file 8. — The main figures in this paper just zoom in on the key sites of selection. These PDFs are also available at https://github.com/jbloomlab/map_flu_serum_Perth2009_H3_HA/tree/master/results/avgdiffsel/full_logo_plots. [file elife-49324-supp8.zip › Supplementary_file_8/ferret-Pitt-2-postinf_diffsel.pdf]

differential selection = 15

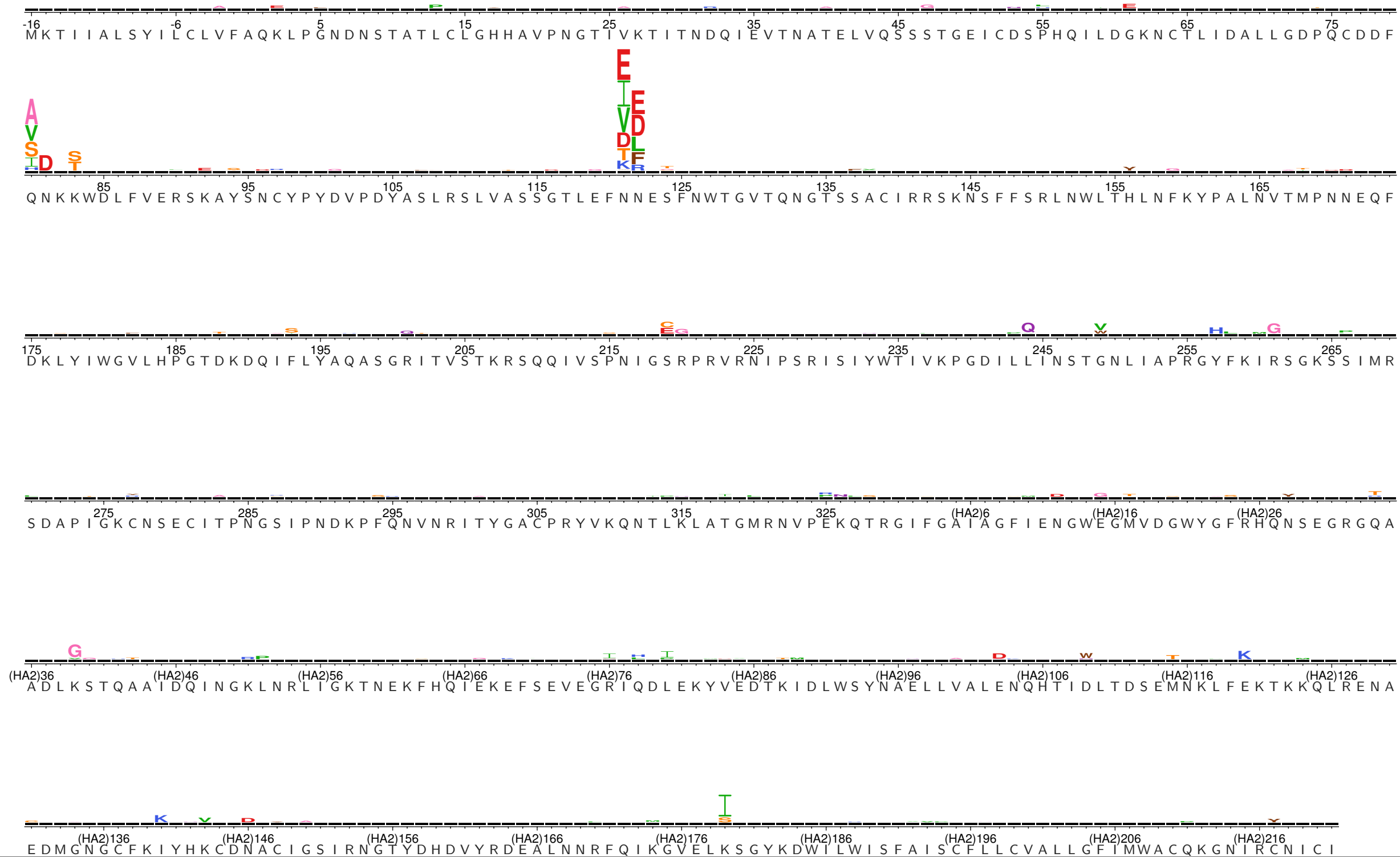

Supplement: Supplementary file 8. — The main figures in this paper just zoom in on the key sites of selection. These PDFs are also available at https://github.com/jbloomlab/map_flu_serum_Perth2009_H3_HA/tree/master/results/avgdiffsel/full_logo_plots. [file elife-49324-supp8.zip › Supplementary_file_8/2009-age-65-with-hi-4F03_diffsel.pdf]

differential selection = 2

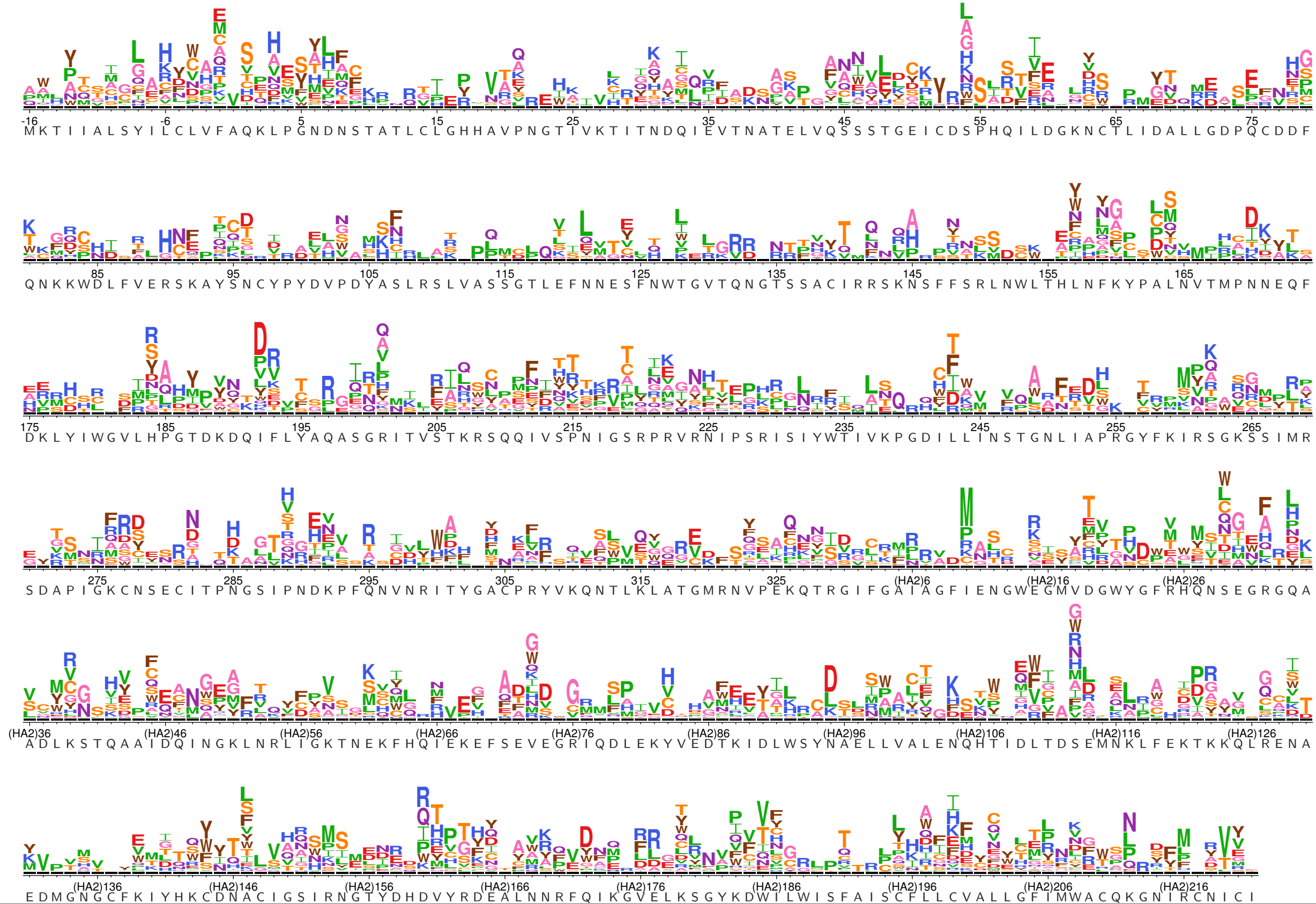

Supplement: Supplementary file 8. — The main figures in this paper just zoom in on the key sites of selection. These PDFs are also available at https://github.com/jbloomlab/map_flu_serum_Perth2009_H3_HA/tree/master/results/avgdiffsel/full_logo_plots. [file elife-49324-supp8.zip › Supplementary_file_8/ferret-Pitt-3-preinf_diffsel.pdf]

differential selection = 43

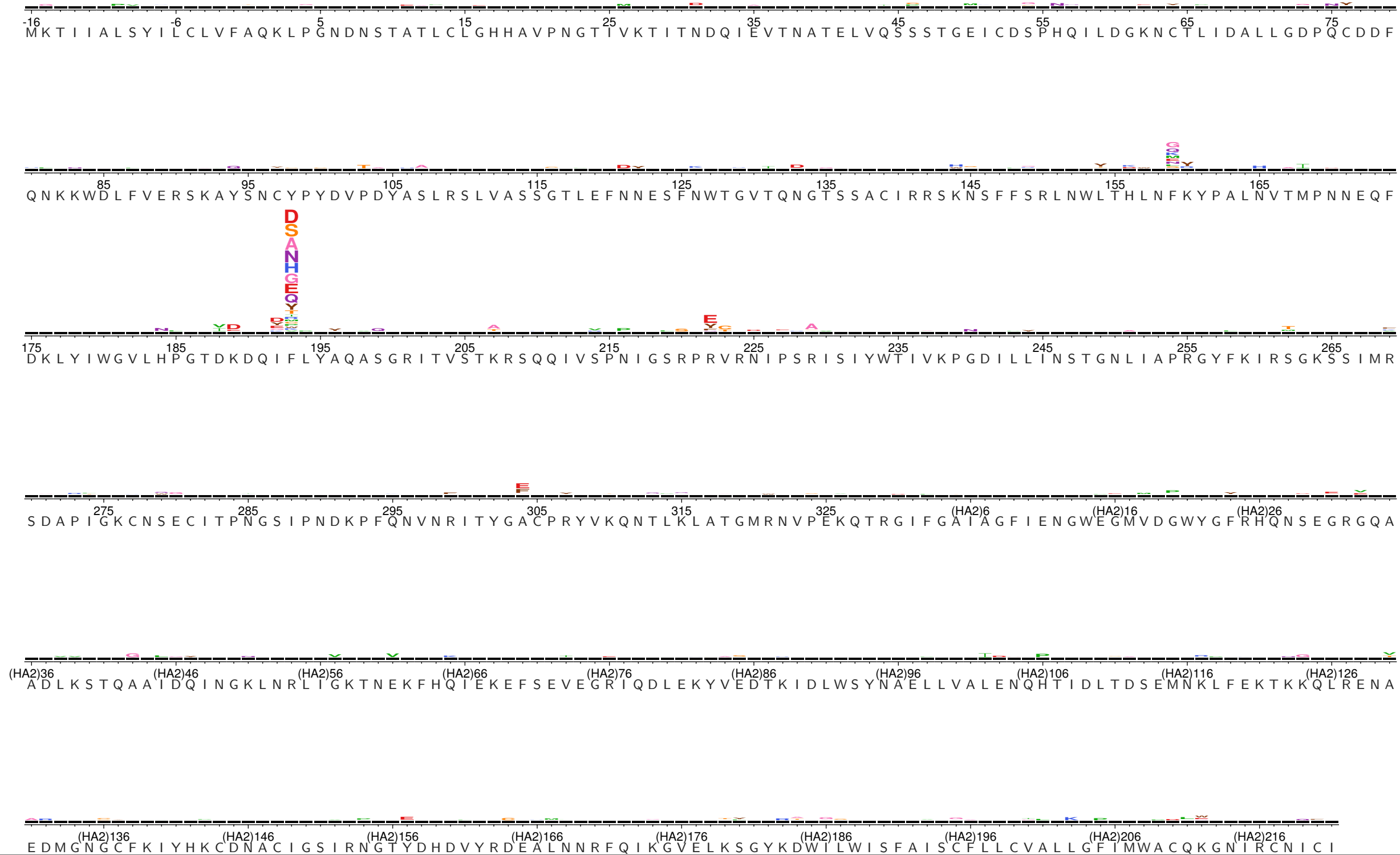

Supplement: Supplementary file 8. — The main figures in this paper just zoom in on the key sites of selection. These PDFs are also available at https://github.com/jbloomlab/map_flu_serum_Perth2009_H3_HA/tree/master/results/avgdiffsel/full_logo_plots. [file elife-49324-supp8.zip › Supplementary_file_8/antibody-4C01_diffsel.pdf]

differential selection = 13

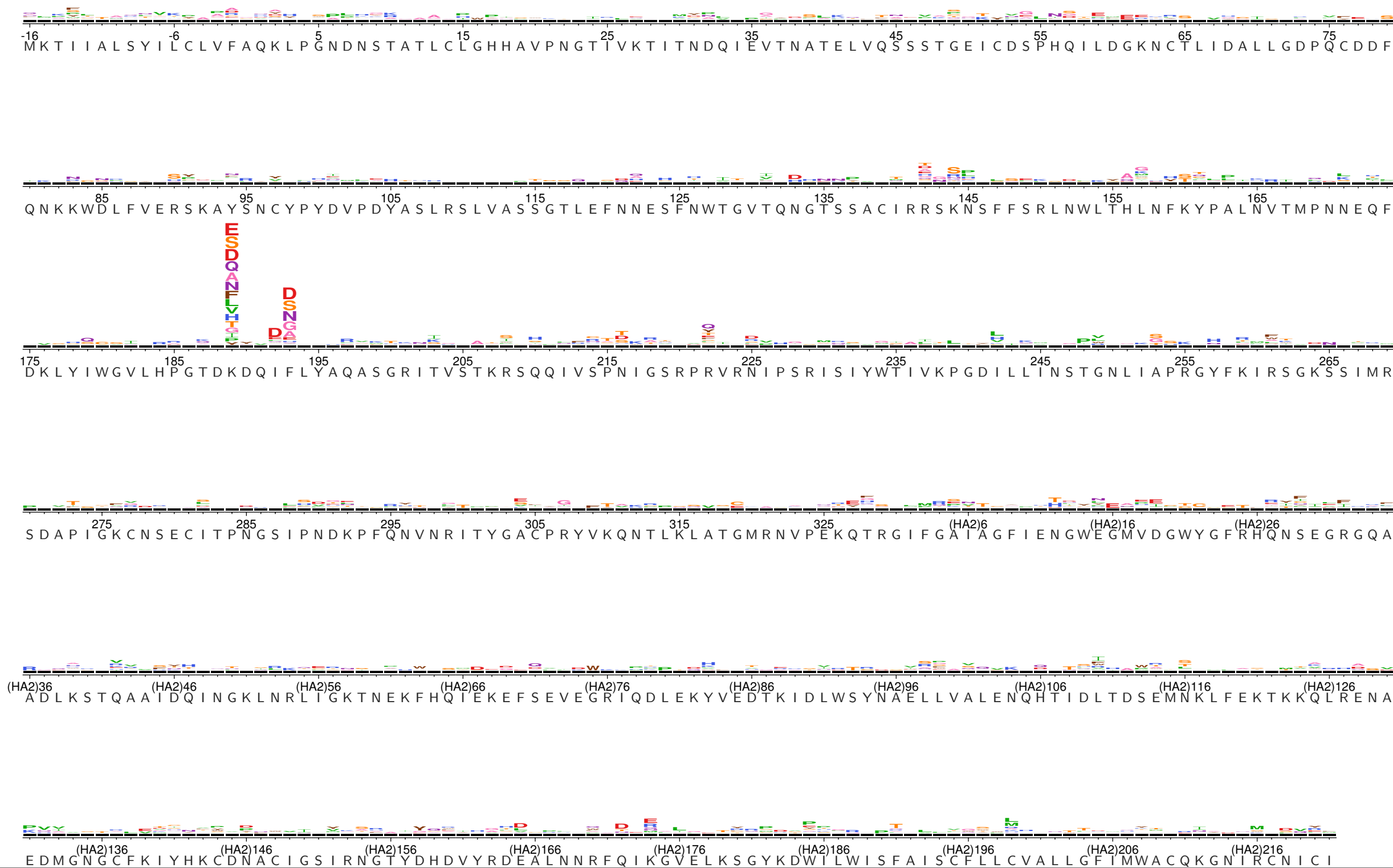

Supplement: Supplementary file 8. — The main figures in this paper just zoom in on the key sites of selection. These PDFs are also available at https://github.com/jbloomlab/map_flu_serum_Perth2009_H3_HA/tree/master/results/avgdiffsel/full_logo_plots. [file elife-49324-supp8.zip › Supplementary_file_8/ferret-Pitt-3-postinf_diffsel.pdf]

differential selection = 12

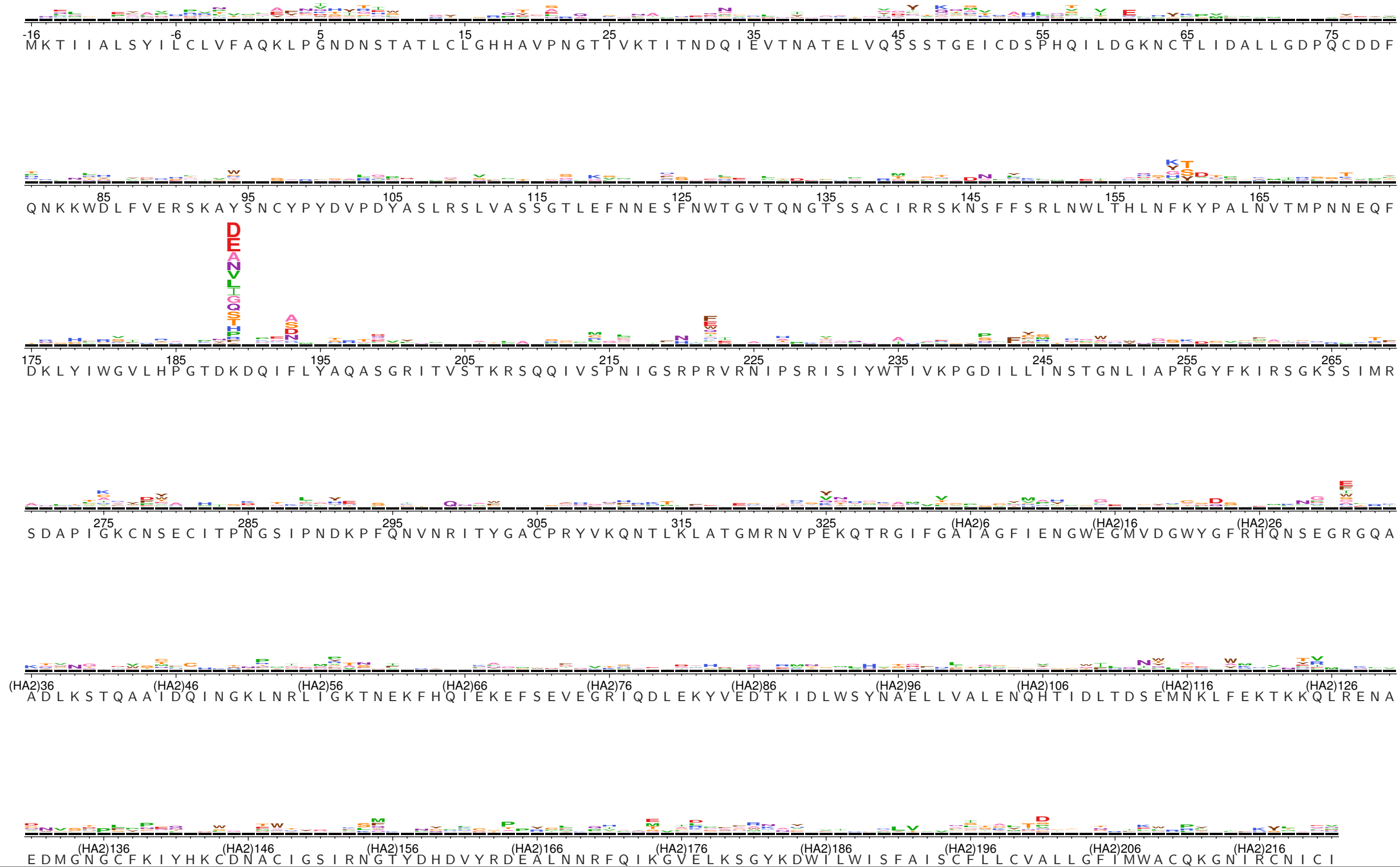

Supplement: Supplementary file 8. — The main figures in this paper just zoom in on the key sites of selection. These PDFs are also available at https://github.com/jbloomlab/map_flu_serum_Perth2009_H3_HA/tree/master/results/avgdiffsel/full_logo_plots. [file elife-49324-supp8.zip › Supplementary_file_8/2015-age-48-vacc_diffsel.pdf]

differential selection = 14

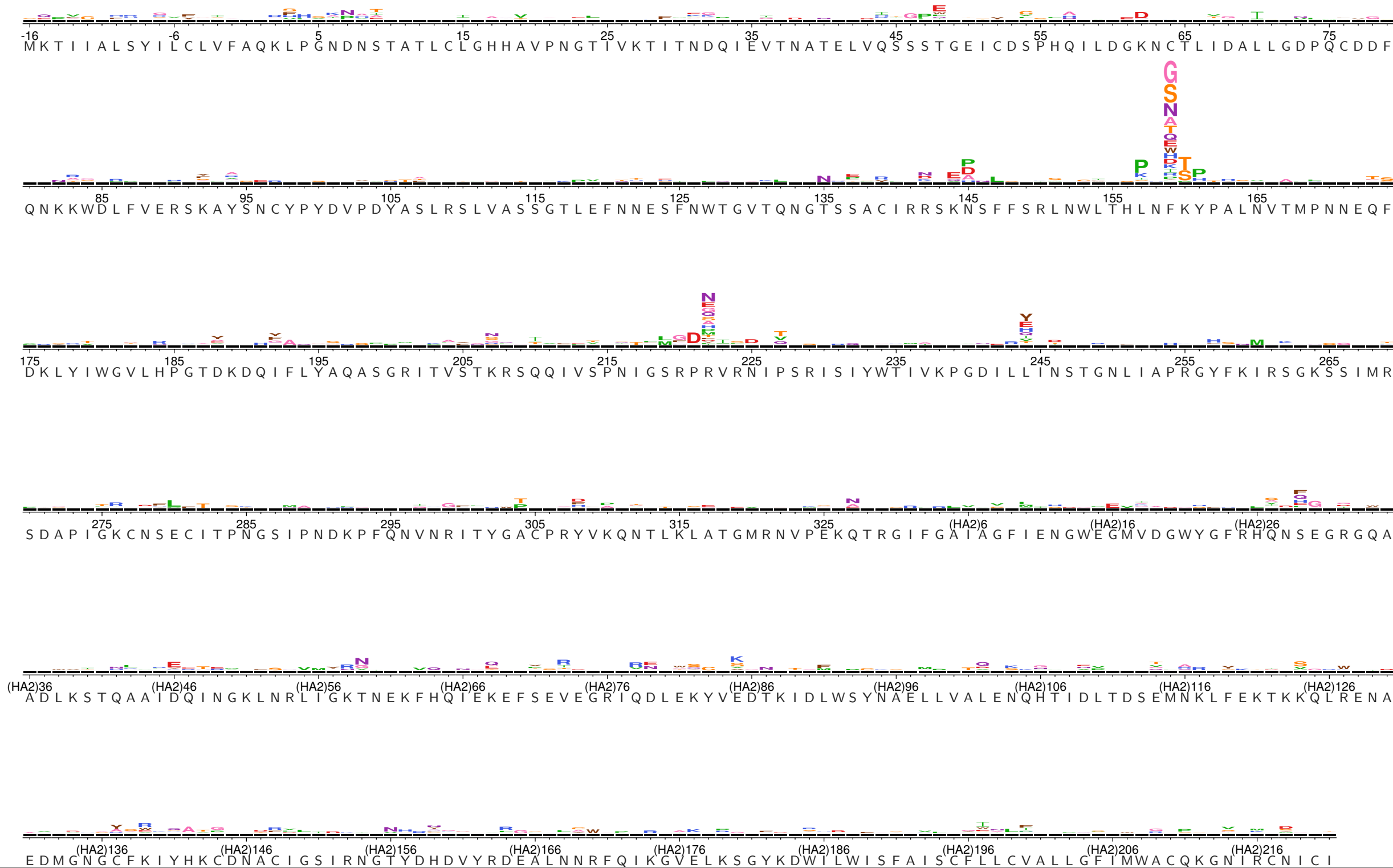

Supplement: Supplementary file 8. — The main figures in this paper just zoom in on the key sites of selection. These PDFs are also available at https://github.com/jbloomlab/map_flu_serum_Perth2009_H3_HA/tree/master/results/avgdiffsel/full_logo_plots. [file elife-49324-supp8.zip › Supplementary_file_8/2009-age-64_diffsel.pdf]

differential selection = 2.1

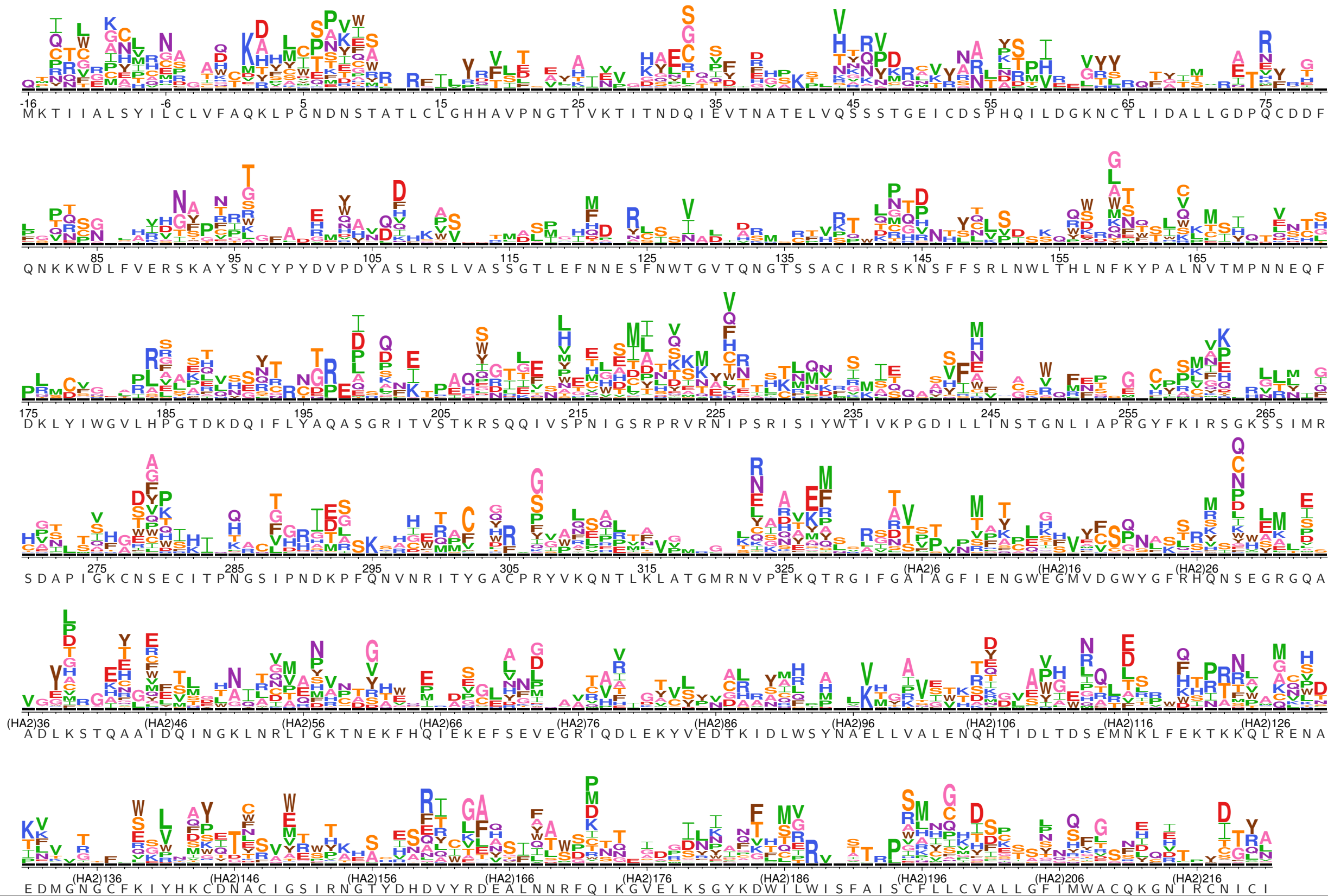

Supplement: Supplementary file 8. — The main figures in this paper just zoom in on the key sites of selection. These PDFs are also available at https://github.com/jbloomlab/map_flu_serum_Perth2009_H3_HA/tree/master/results/avgdiffsel/full_logo_plots. [file elife-49324-supp8.zip › Supplementary_file_8/2015-age-49-prevacc_diffsel.pdf]

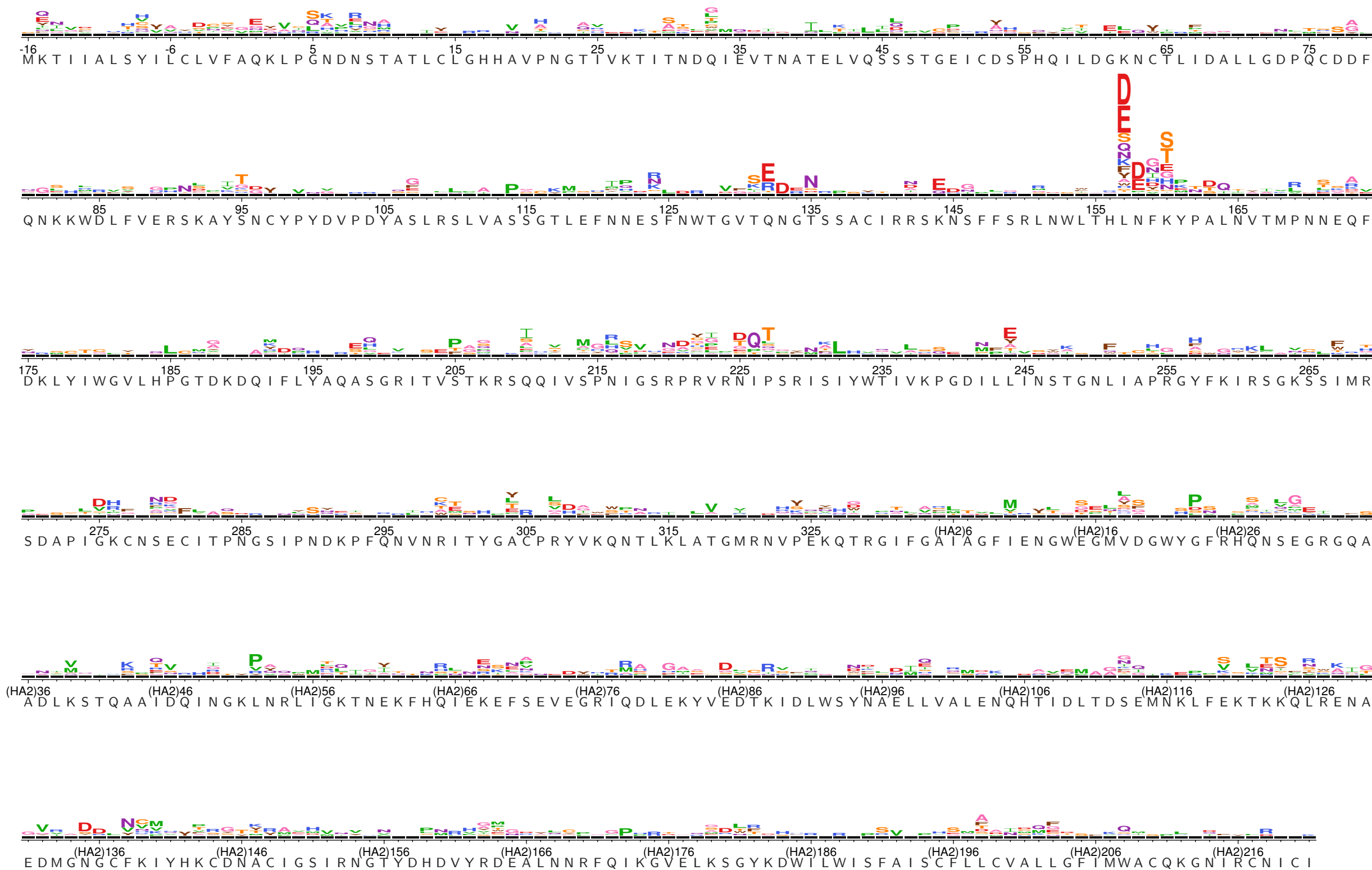

Supplement: Supplementary file 8. — The main figures in this paper just zoom in on the key sites of selection. These PDFs are also available at https://github.com/jbloomlab/map_flu_serum_Perth2009_H3_HA/tree/master/results/avgdiffsel/full_logo_plots. [file elife-49324-supp8.zip › Supplementary_file_8/2009-age-53a_diffsel.pdf]

differential selection = 13

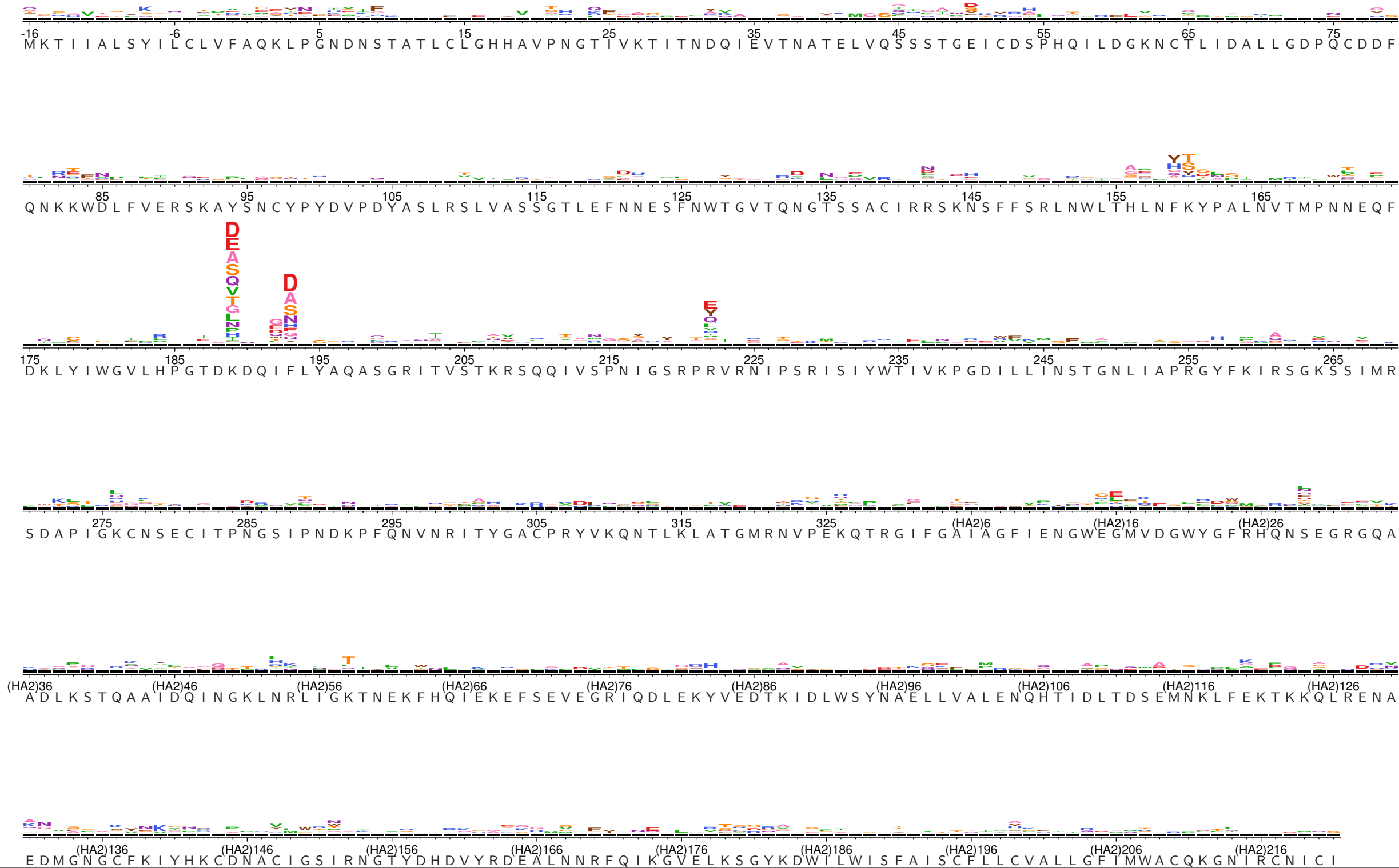

Supplement: Supplementary file 8. — The main figures in this paper just zoom in on the key sites of selection. These PDFs are also available at https://github.com/jbloomlab/map_flu_serum_Perth2009_H3_HA/tree/master/results/avgdiffsel/full_logo_plots. [file elife-49324-supp8.zip › Supplementary_file_8/ferret-Pitt-1-postinf_diffsel.pdf]

differential selection = 2.5

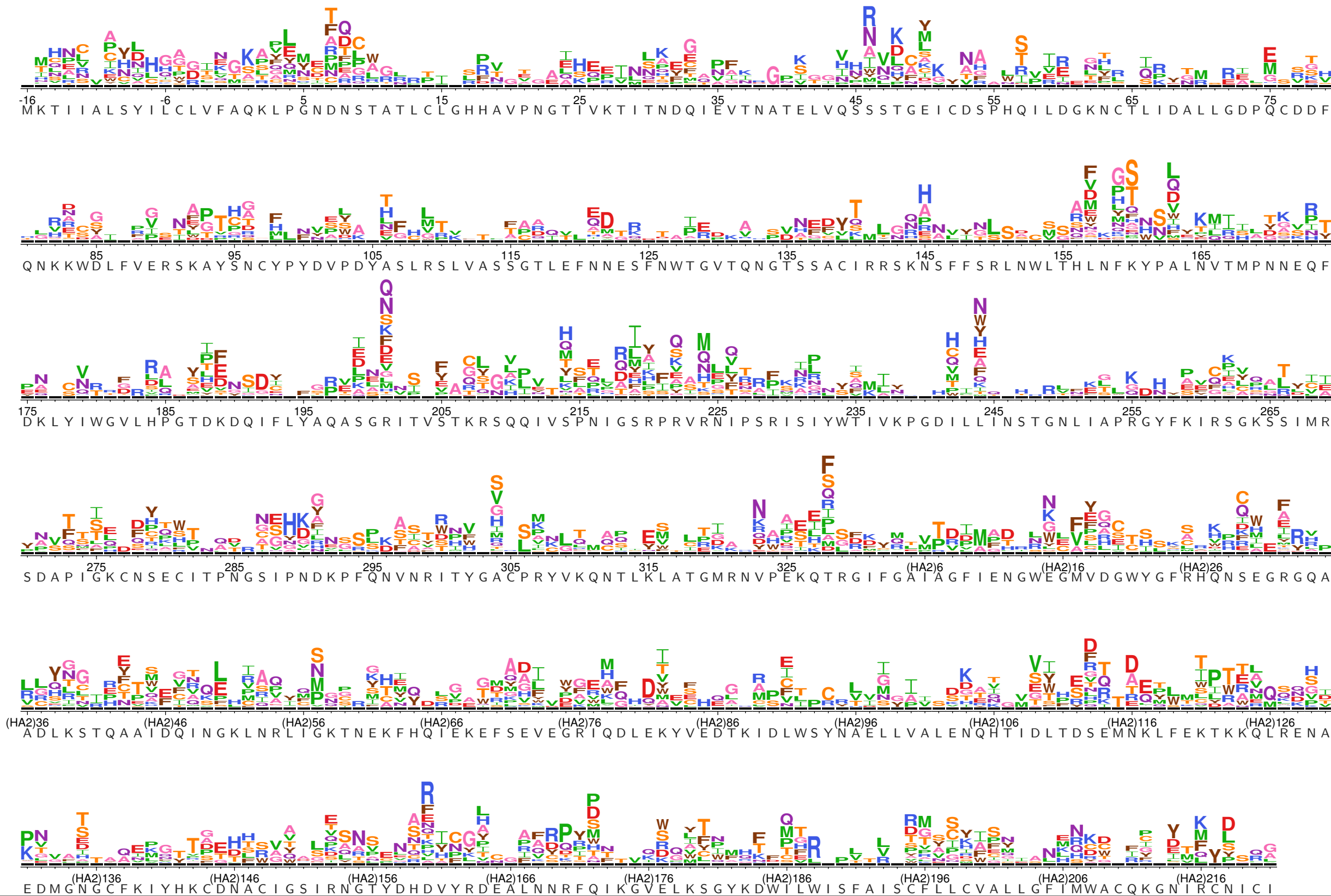

Supplement: Supplementary file 8. — The main figures in this paper just zoom in on the key sites of selection. These PDFs are also available at https://github.com/jbloomlab/map_flu_serum_Perth2009_H3_HA/tree/master/results/avgdiffsel/full_logo_plots. [file elife-49324-supp8.zip › Supplementary_file_8/2015-age-48-prevacc_diffsel.pdf]

differential selection = 24

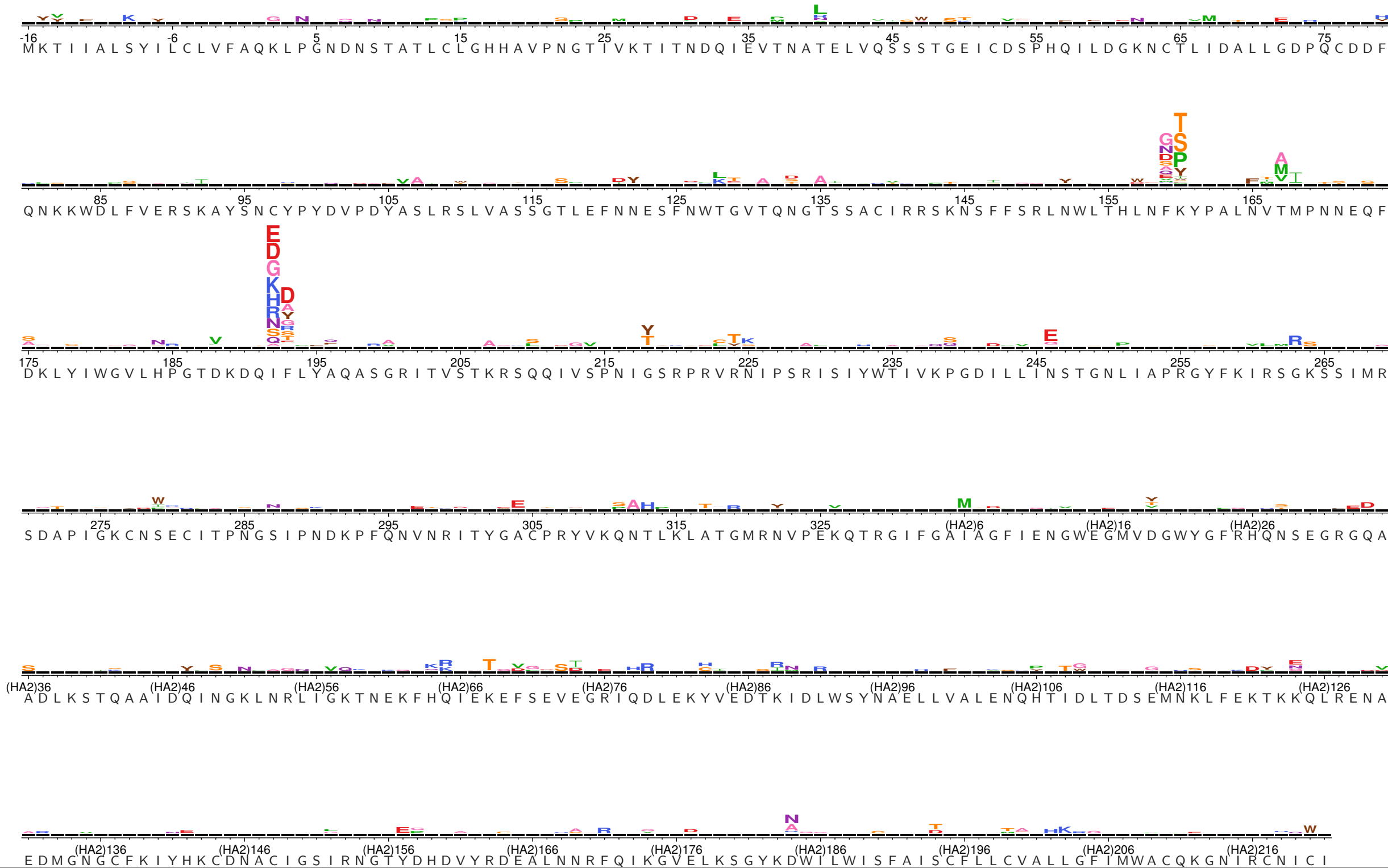

Supplement: Supplementary file 8. — The main figures in this paper just zoom in on the key sites of selection. These PDFs are also available at https://github.com/jbloomlab/map_flu_serum_Perth2009_H3_HA/tree/master/results/avgdiffsel/full_logo_plots. [file elife-49324-supp8.zip › Supplementary_file_8/antibody-3C04_diffsel.pdf]

differential selection = 17

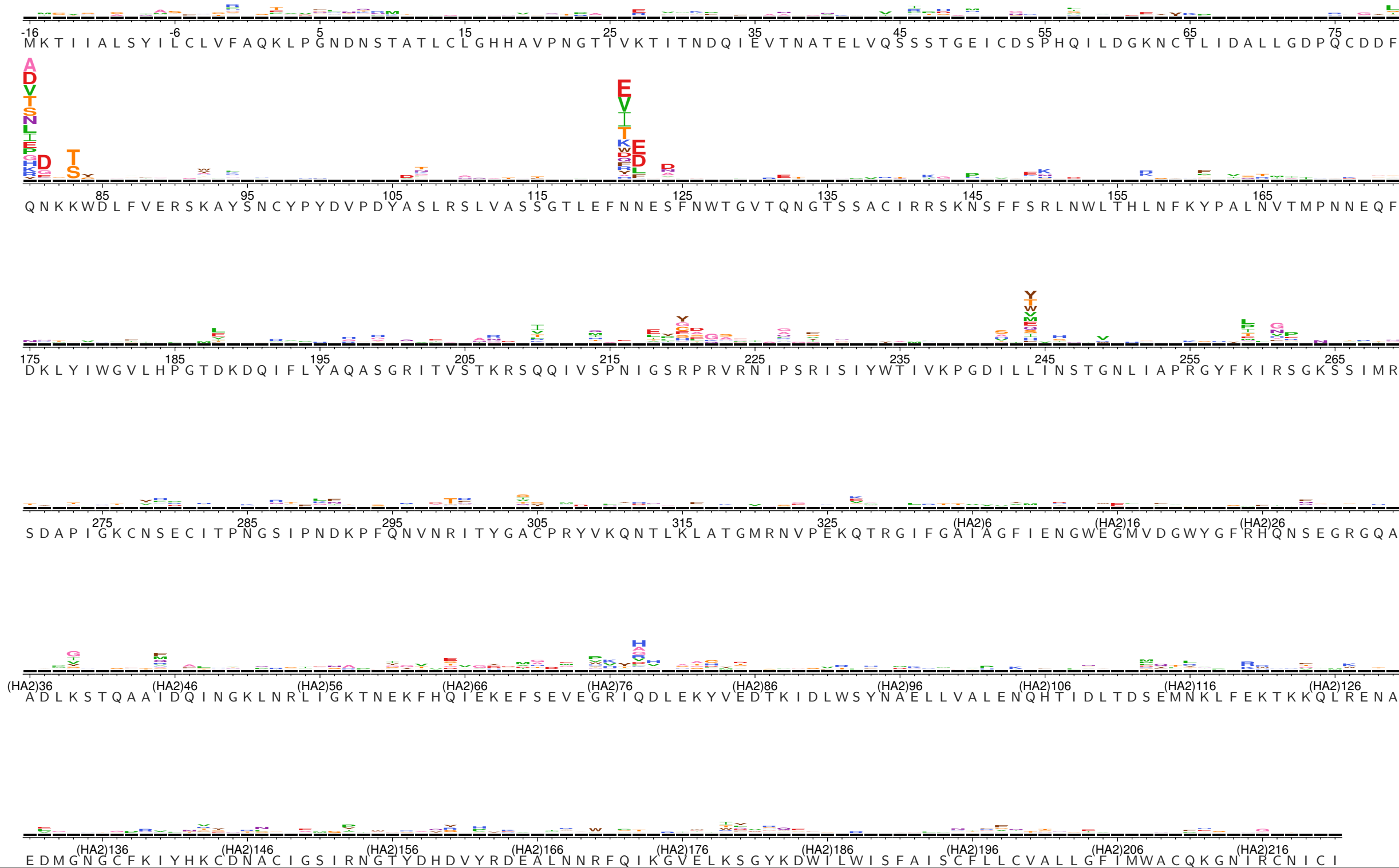

Supplement: Supplementary file 8. — The main figures in this paper just zoom in on the key sites of selection. These PDFs are also available at https://github.com/jbloomlab/map_flu_serum_Perth2009_H3_HA/tree/master/results/avgdiffsel/full_logo_plots. [file elife-49324-supp8.zip › Supplementary_file_8/antibody-4F03_diffsel.pdf]

differential selection = 8.2

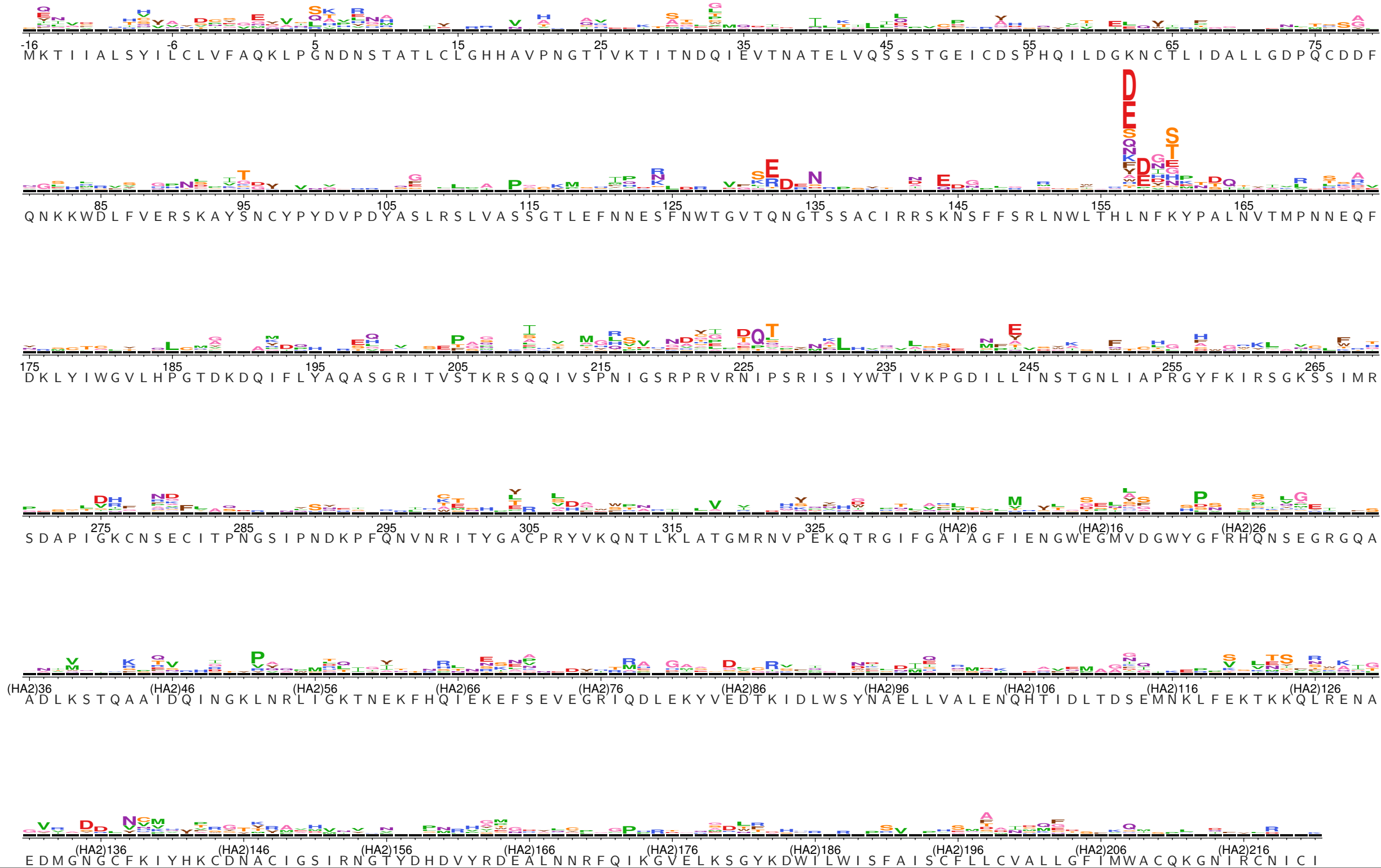

Supplement: Supplementary file 8. — The main figures in this paper just zoom in on the key sites of selection. These PDFs are also available at https://github.com/jbloomlab/map_flu_serum_Perth2009_H3_HA/tree/master/results/avgdiffsel/full_logo_plots. [file elife-49324-supp8.zip › Supplementary_file_8/2009-age-53_diffsel.pdf]

differential selection = 6.1

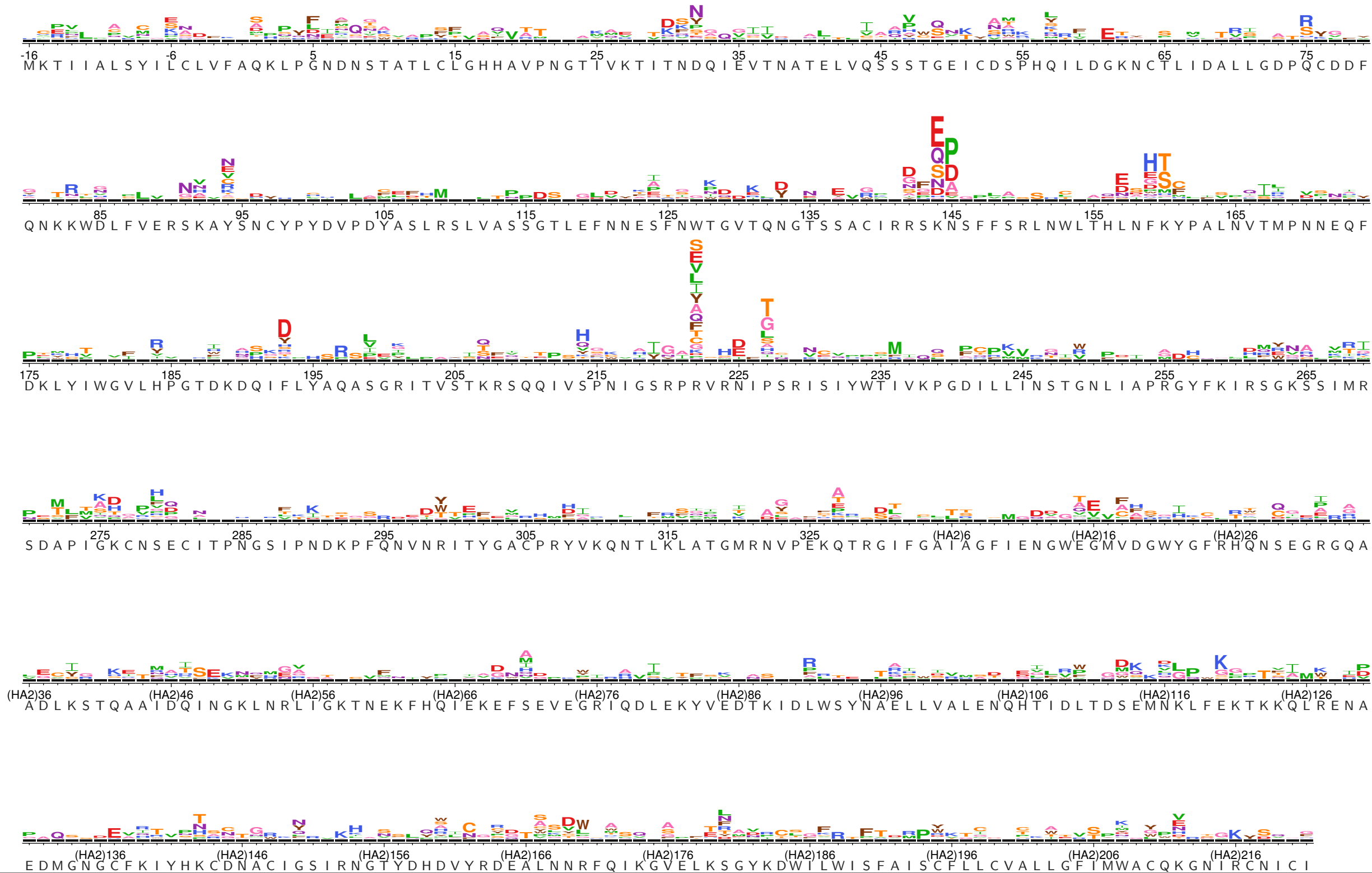

Supplement: Supplementary file 8. — The main figures in this paper just zoom in on the key sites of selection. These PDFs are also available at https://github.com/jbloomlab/map_flu_serum_Perth2009_H3_HA/tree/master/results/avgdiffsel/full_logo_plots. [file elife-49324-supp8.zip › Supplementary_file_8/2015-age-29-vacc_diffsel.pdf]

differential selection = 2.6

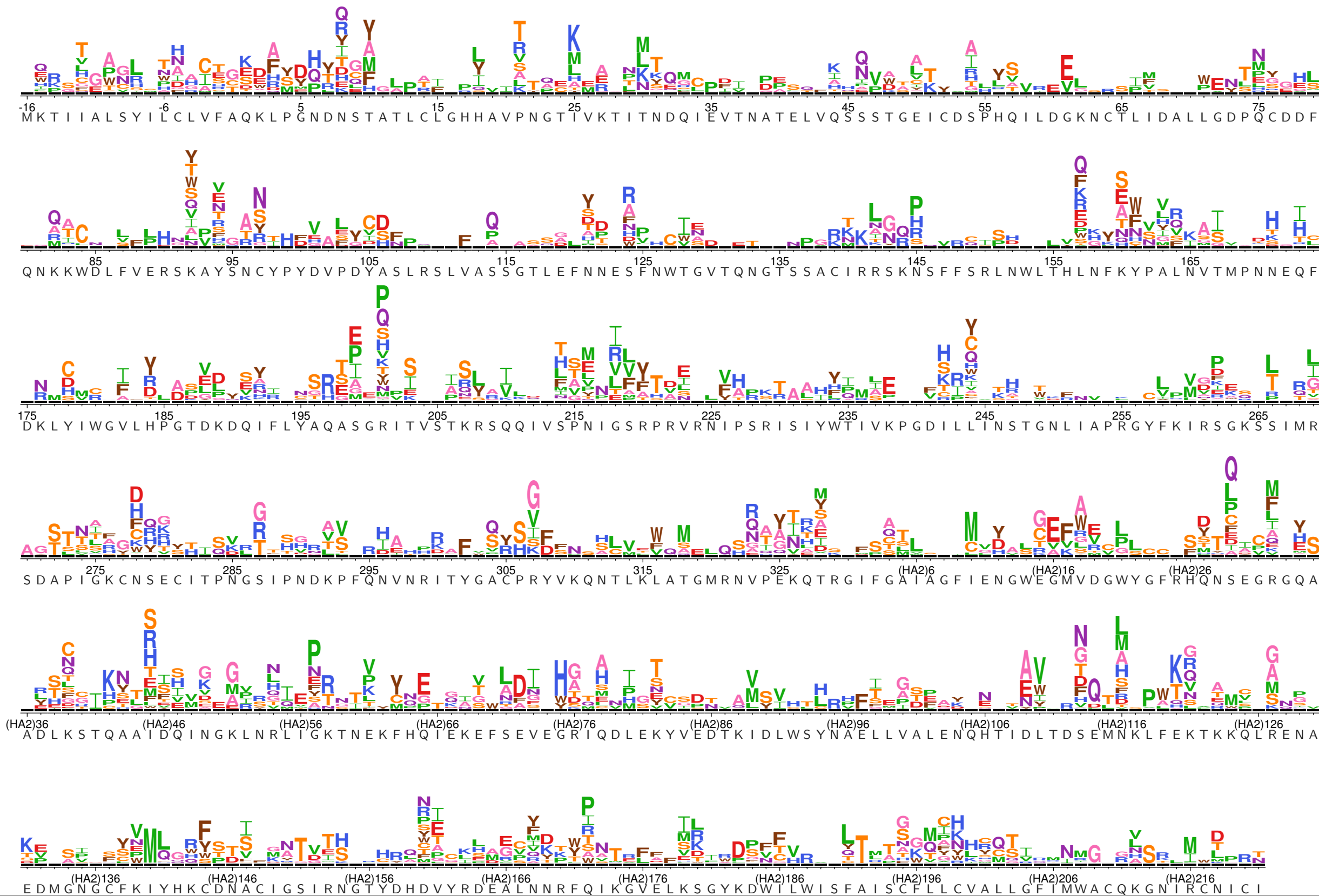

Supplement: Supplementary file 8. — The main figures in this paper just zoom in on the key sites of selection. These PDFs are also available at https://github.com/jbloomlab/map_flu_serum_Perth2009_H3_HA/tree/master/results/avgdiffsel/full_logo_plots. [file elife-49324-supp8.zip › Supplementary_file_8/2015-age-49-vacc_diffsel.pdf]

differential selection = 9.4

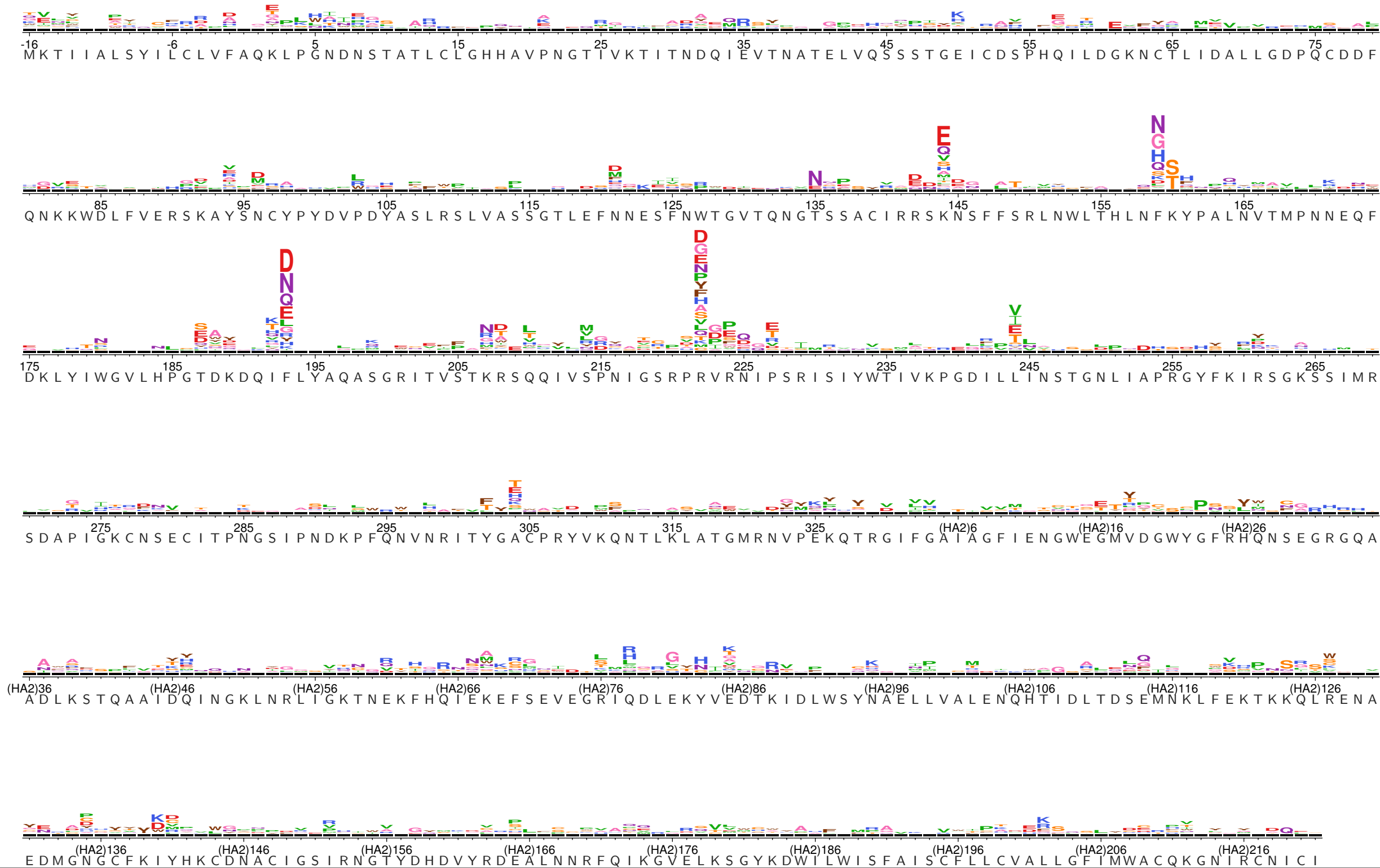

Supplement: Supplementary file 8. — The main figures in this paper just zoom in on the key sites of selection. These PDFs are also available at https://github.com/jbloomlab/map_flu_serum_Perth2009_H3_HA/tree/master/results/avgdiffsel/full_logo_plots. [file elife-49324-supp8.zip › Supplementary_file_8/2010-age-21_diffsel.pdf]

differential selection = 10

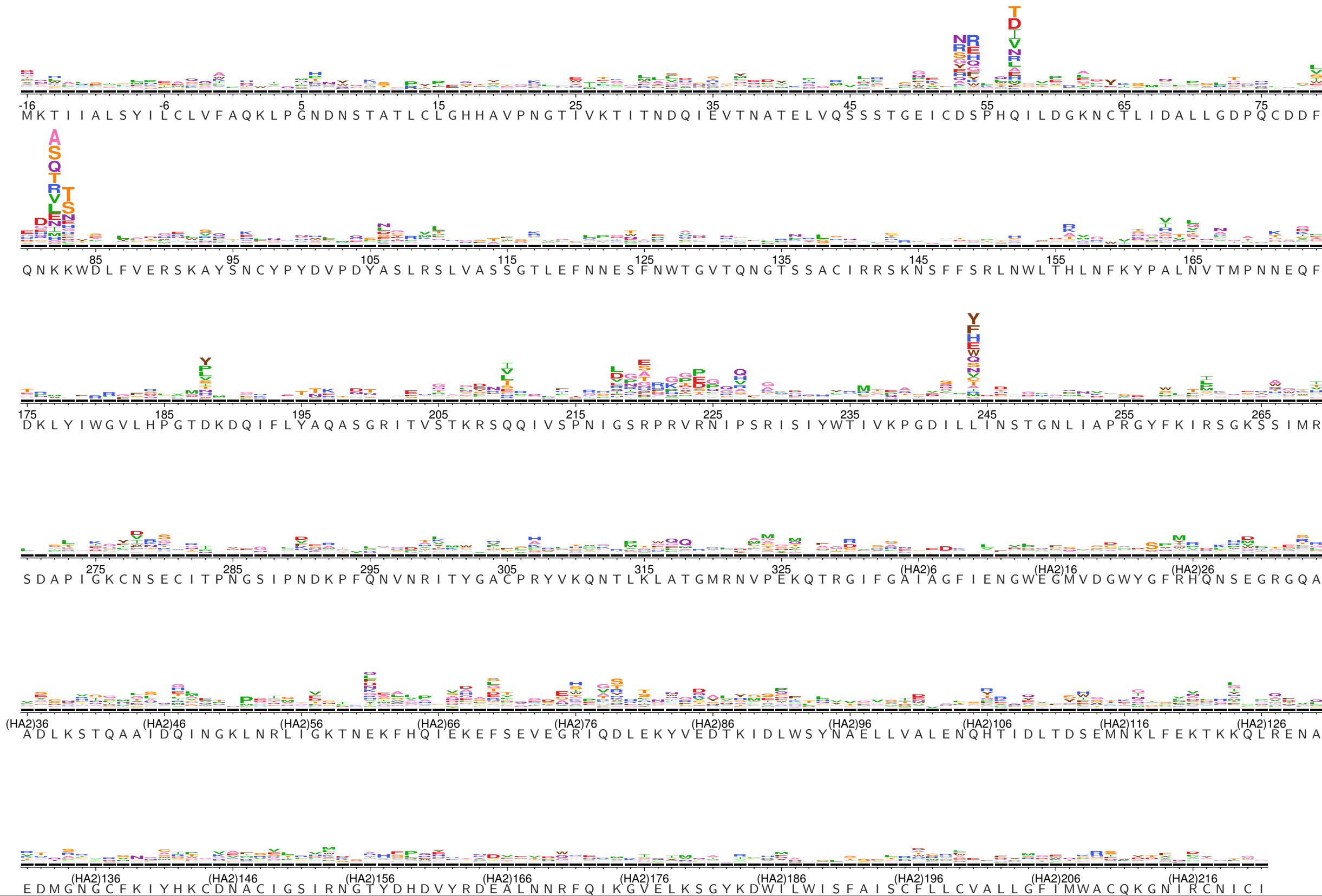

Supplement: Supplementary file 8. — The main figures in this paper just zoom in on the key sites of selection. These PDFs are also available at https://github.com/jbloomlab/map_flu_serum_Perth2009_H3_HA/tree/master/results/avgdiffsel/full_logo_plots. [file elife-49324-supp8.zip › Supplementary_file_8/antibody-1C04_diffsel.pdf]

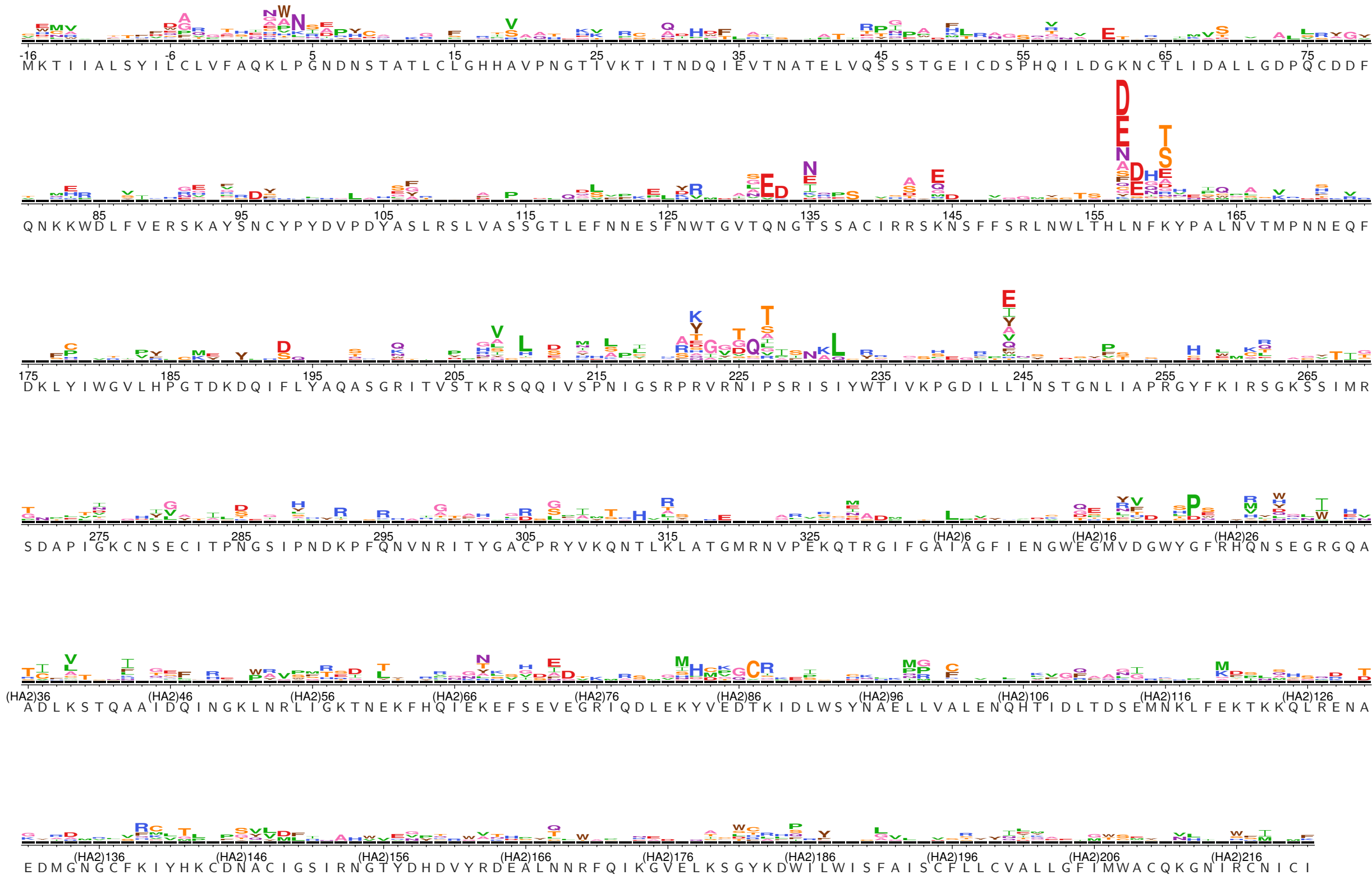

Supplement: Supplementary file 8. — The main figures in this paper just zoom in on the key sites of selection. These PDFs are also available at https://github.com/jbloomlab/map_flu_serum_Perth2009_H3_HA/tree/master/results/avgdiffsel/full_logo_plots. [file elife-49324-supp8.zip › Supplementary_file_8/2009-age-53b_diffsel.pdf]

differential selection = 14

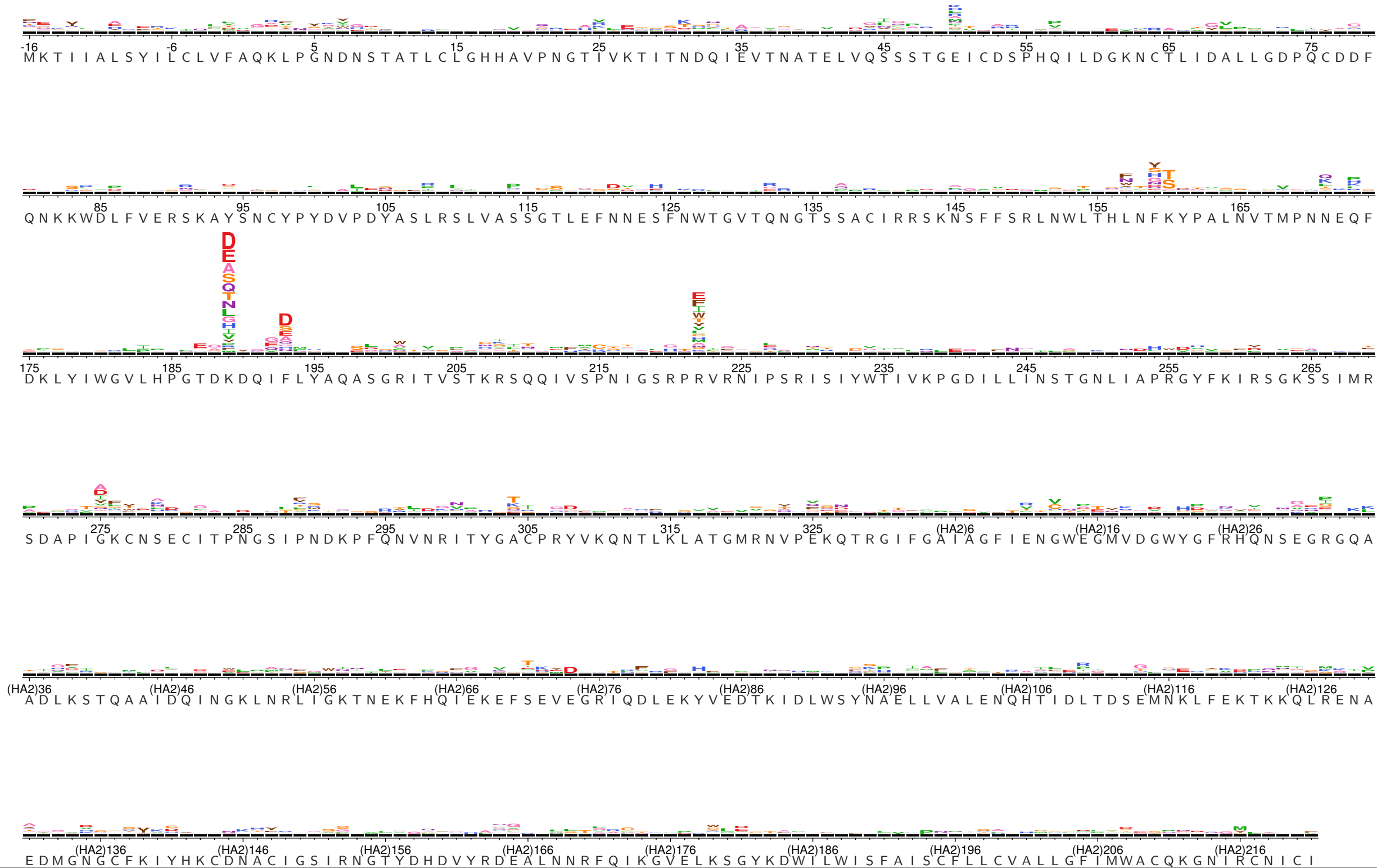

Supplement: Supplementary file 8. — The main figures in this paper just zoom in on the key sites of selection. These PDFs are also available at https://github.com/jbloomlab/map_flu_serum_Perth2009_H3_HA/tree/master/results/avgdiffsel/full_logo_plots. [file elife-49324-supp8.zip › Supplementary_file_8/ferret-WHO-Victoria2011_diffsel.pdf]

differential selection = 15

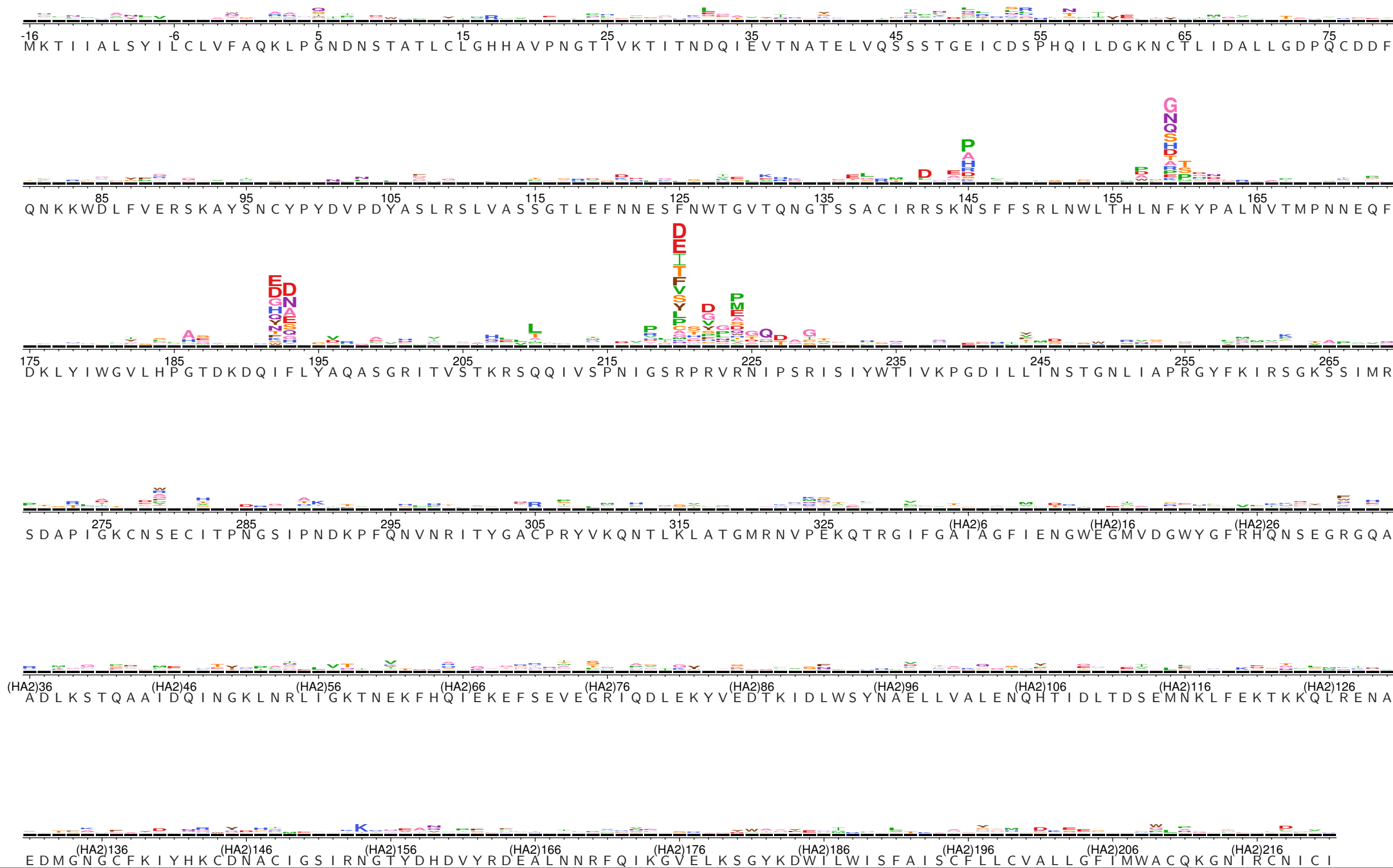

Supplement: Supplementary file 8. — The main figures in this paper just zoom in on the key sites of selection. These PDFs are also available at https://github.com/jbloomlab/map_flu_serum_Perth2009_H3_HA/tree/master/results/avgdiffsel/full_logo_plots. [file elife-49324-supp8.zip › Supplementary_file_8/2015-age-25-prevacc_diffsel.pdf]

differential selection = 13

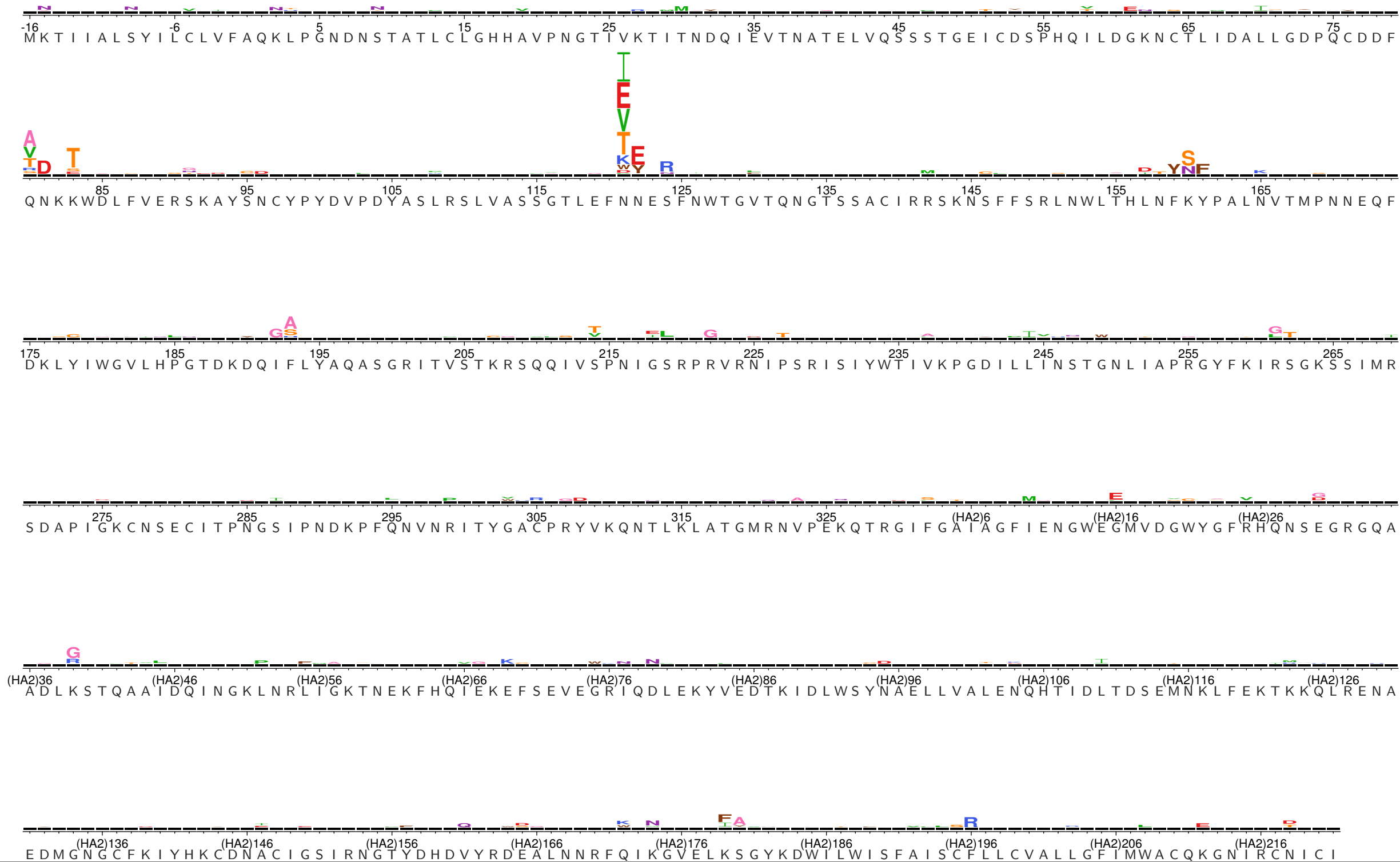

Supplement: Supplementary file 8. — The main figures in this paper just zoom in on the key sites of selection. These PDFs are also available at https://github.com/jbloomlab/map_flu_serum_Perth2009_H3_HA/tree/master/results/avgdiffsel/full_logo_plots. [file elife-49324-supp8.zip › Supplementary_file_8/2009-age-65-with-mid-4F03_diffsel.pdf]

differential selection = 9.6

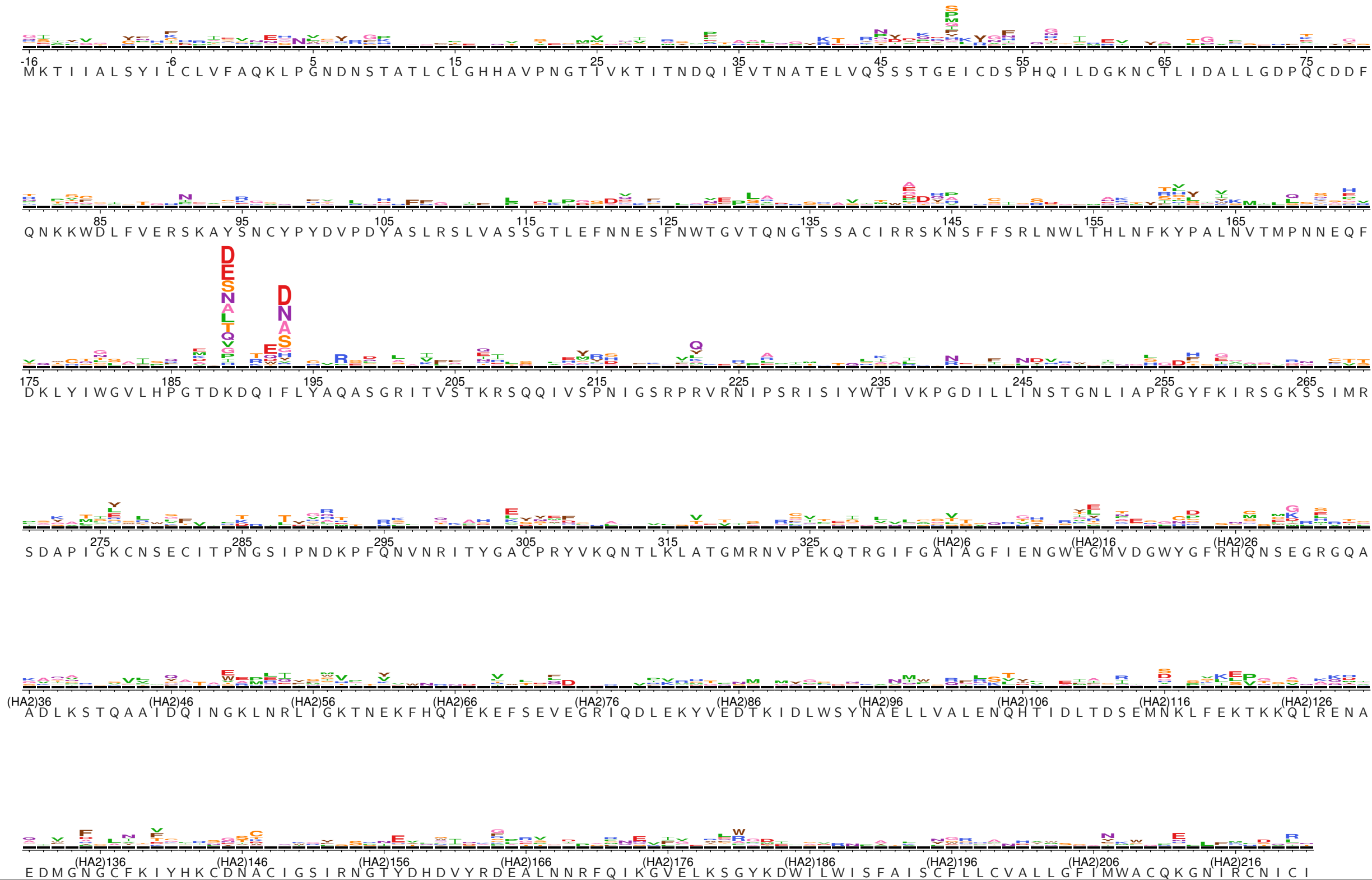

Supplement: Supplementary file 8. — The main figures in this paper just zoom in on the key sites of selection. These PDFs are also available at https://github.com/jbloomlab/map_flu_serum_Perth2009_H3_HA/tree/master/results/avgdiffsel/full_logo_plots. [file elife-49324-supp8.zip › Supplementary_file_8/ferret-WHO-Perth2009_diffsel.pdf]

differential selection = 7.1

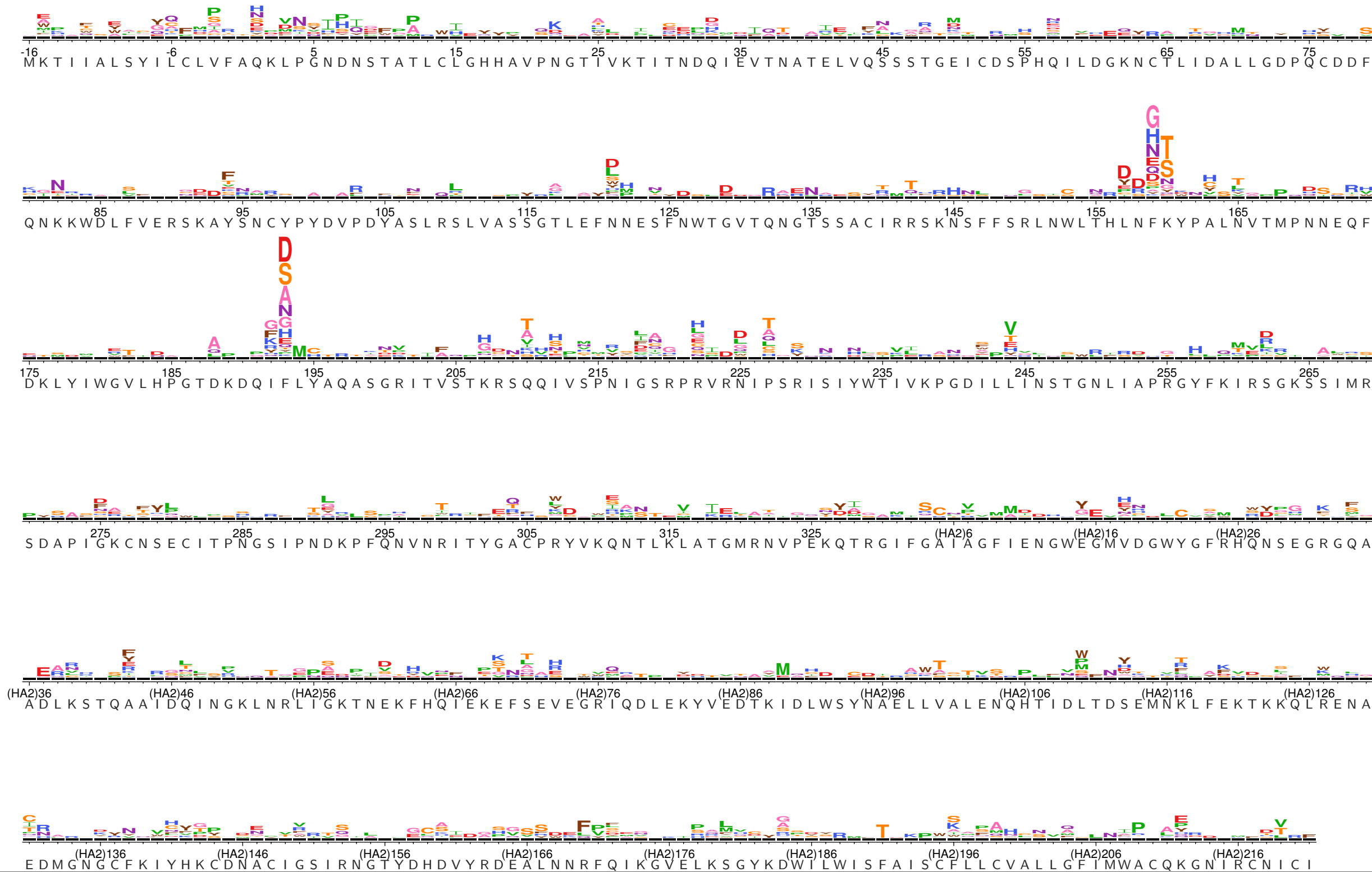

Supplement: Supplementary file 8. — The main figures in this paper just zoom in on the key sites of selection. These PDFs are also available at https://github.com/jbloomlab/map_flu_serum_Perth2009_H3_HA/tree/master/results/avgdiffsel/full_logo_plots. [file elife-49324-supp8.zip › Supplementary_file_8/2009-age-65_diffsel.pdf]

differential selection = 9.6

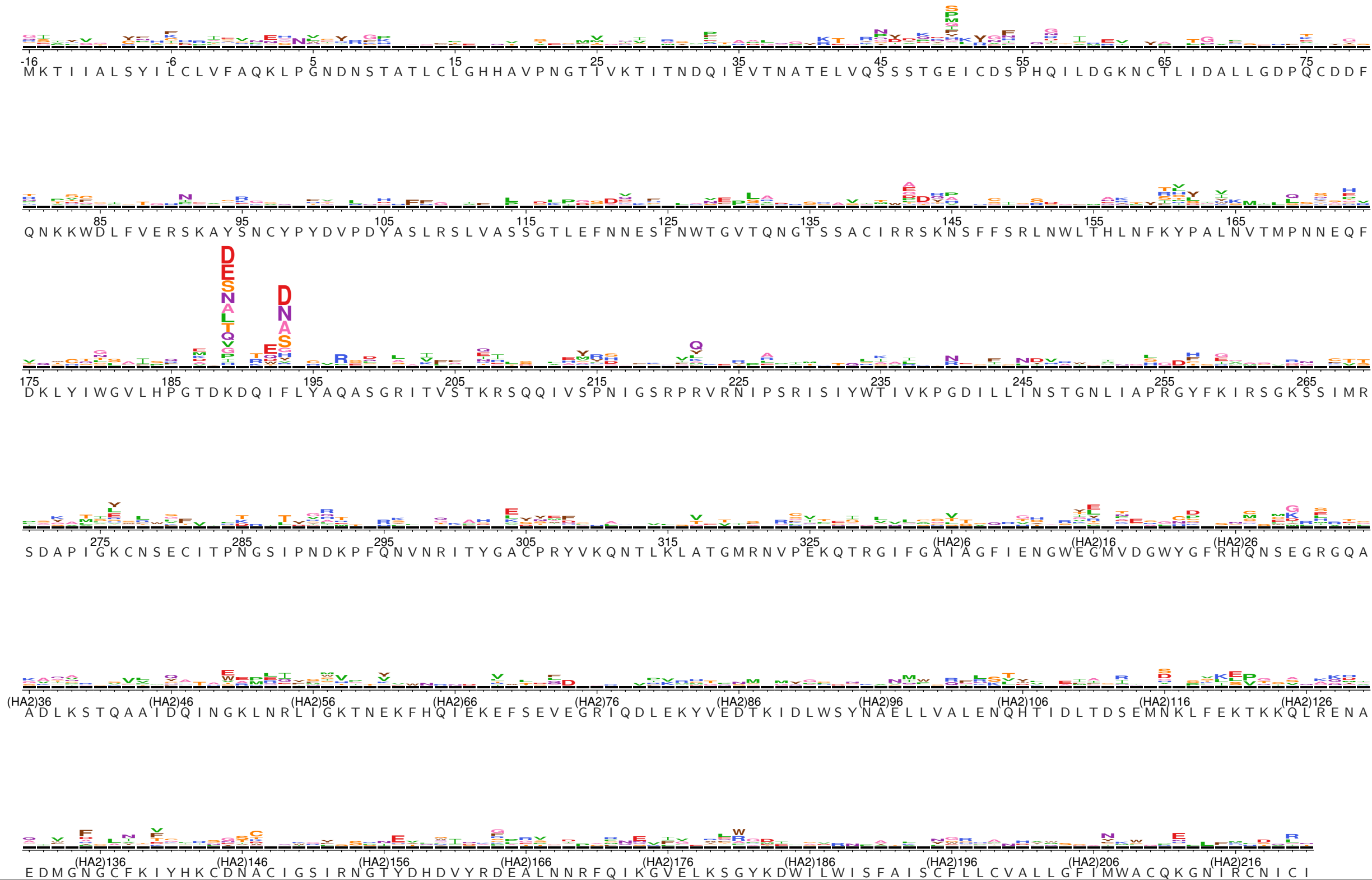

Supplement: Supplementary file 8. — The main figures in this paper just zoom in on the key sites of selection. These PDFs are also available at https://github.com/jbloomlab/map_flu_serum_Perth2009_H3_HA/tree/master/results/avgdiffsel/full_logo_plots. [file elife-49324-supp8.zip › Supplementary_file_8/ferret-WHO_diffsel.pdf]

differential selection = 6.8

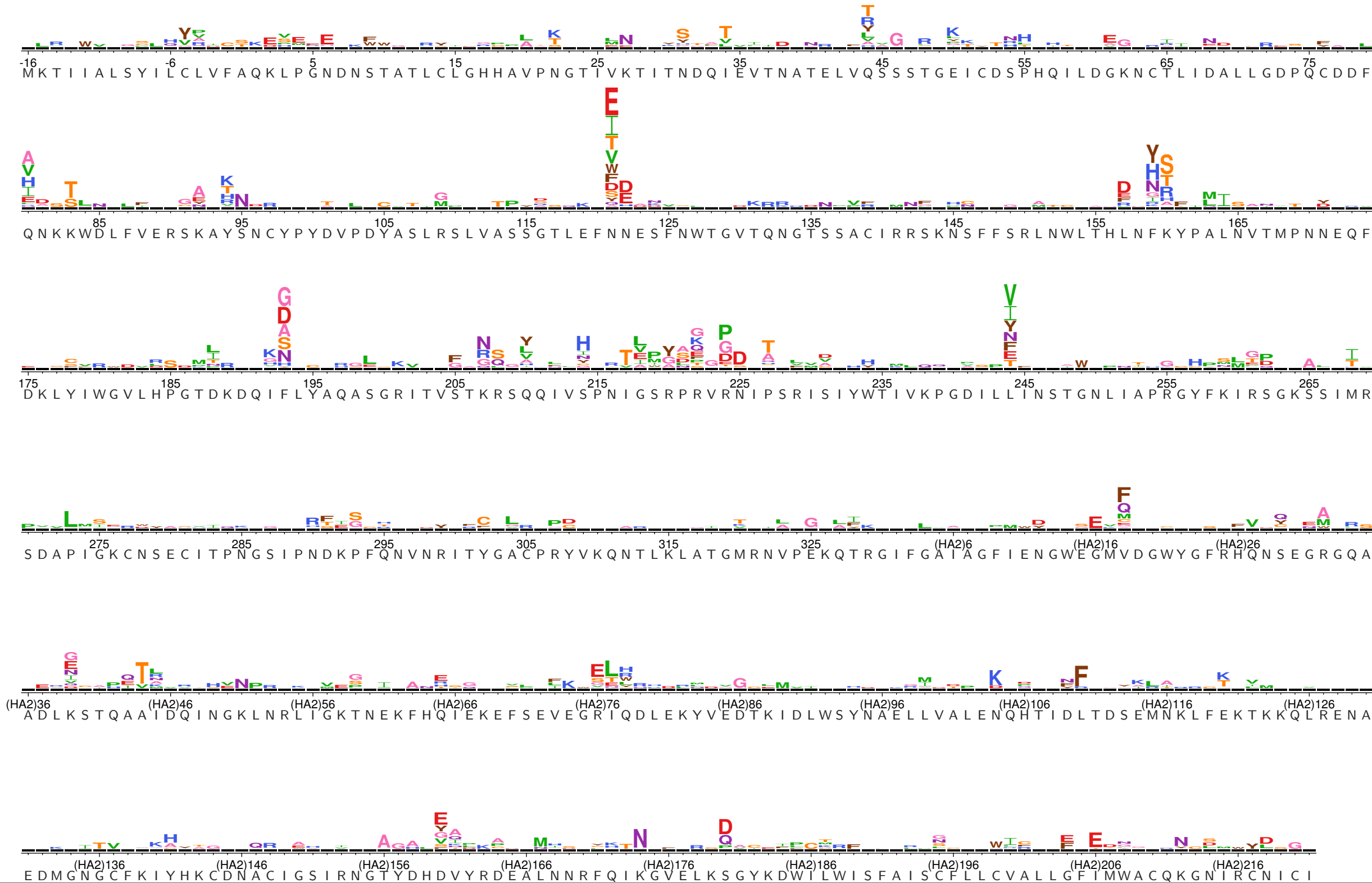

Supplement: Supplementary file 8. — The main figures in this paper just zoom in on the key sites of selection. These PDFs are also available at https://github.com/jbloomlab/map_flu_serum_Perth2009_H3_HA/tree/master/results/avgdiffsel/full_logo_plots. [file elife-49324-supp8.zip › Supplementary_file_8/2009-age-65-with-low-4F03_diffsel.pdf]

differential selection = 1.5

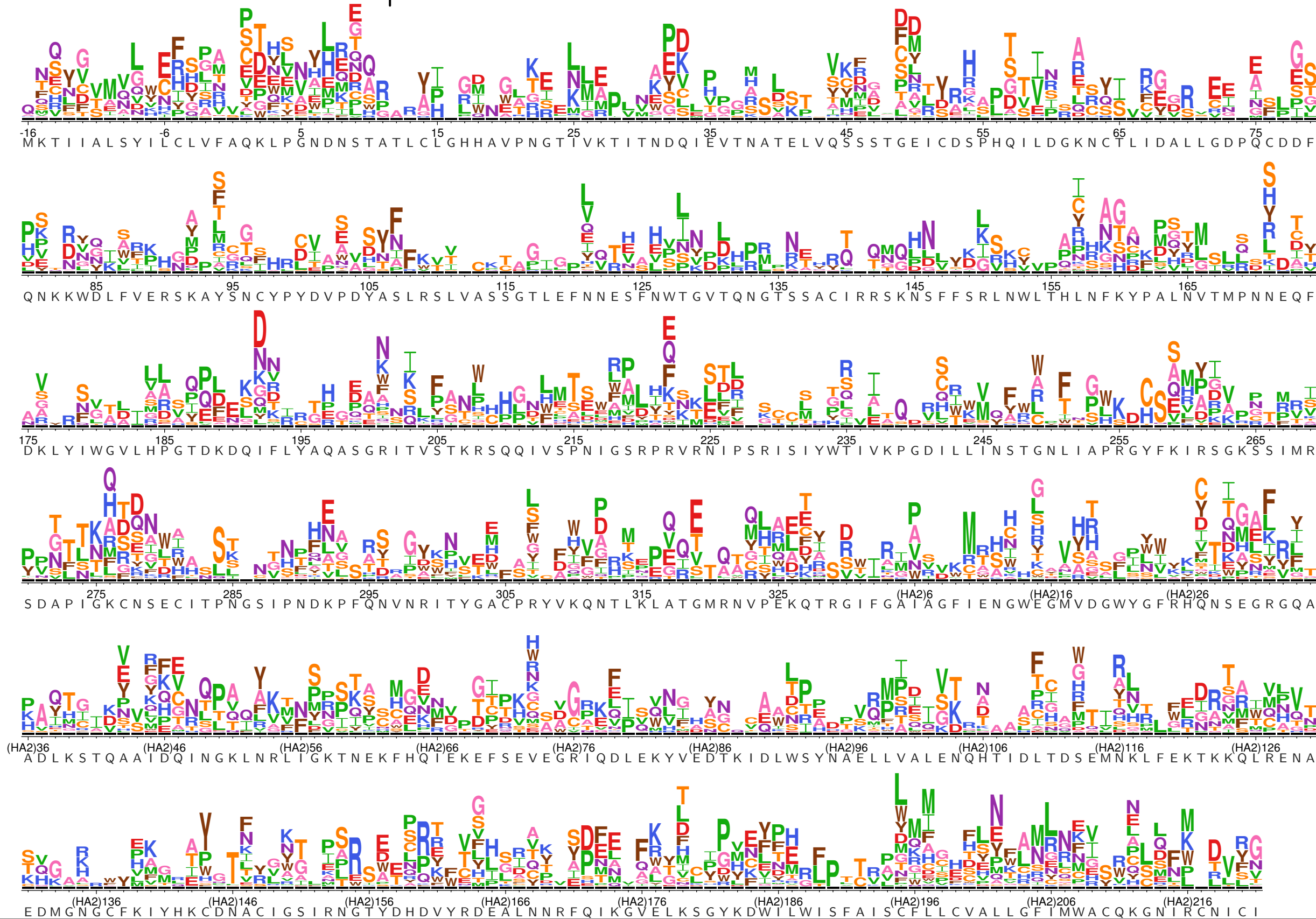

Supplement: Supplementary file 8. — The main figures in this paper just zoom in on the key sites of selection. These PDFs are also available at https://github.com/jbloomlab/map_flu_serum_Perth2009_H3_HA/tree/master/results/avgdiffsel/full_logo_plots. [file elife-49324-supp8.zip › Supplementary_file_8/ferret-Pitt-1-preinf_diffsel.pdf]

differential selection = 16

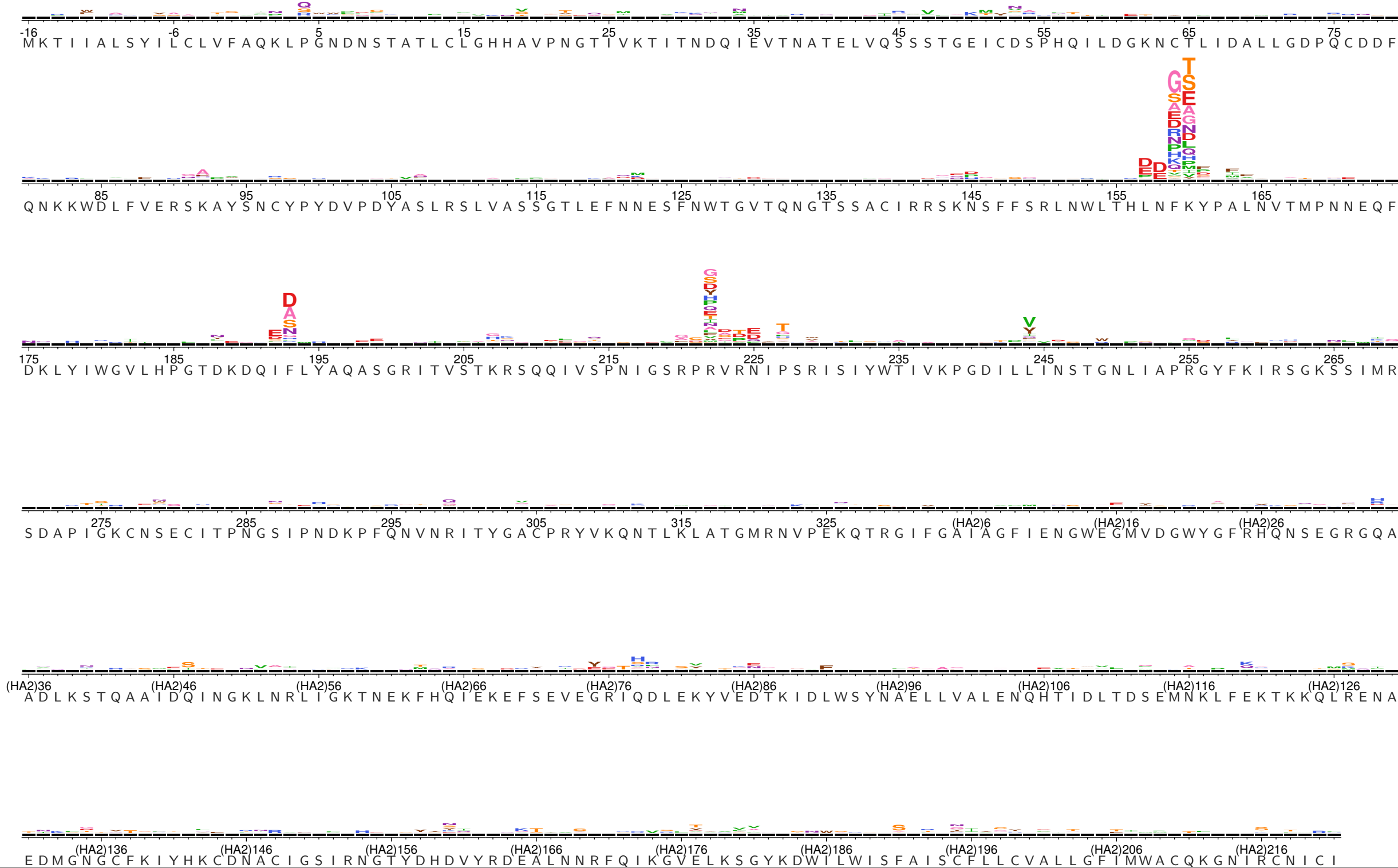

Supplement: Supplementary file 8. — The main figures in this paper just zoom in on the key sites of selection. These PDFs are also available at https://github.com/jbloomlab/map_flu_serum_Perth2009_H3_HA/tree/master/results/avgdiffsel/full_logo_plots. [file elife-49324-supp8.zip › Supplementary_file_8/antibody-5A01_diffsel.pdf]

differential selection = 24

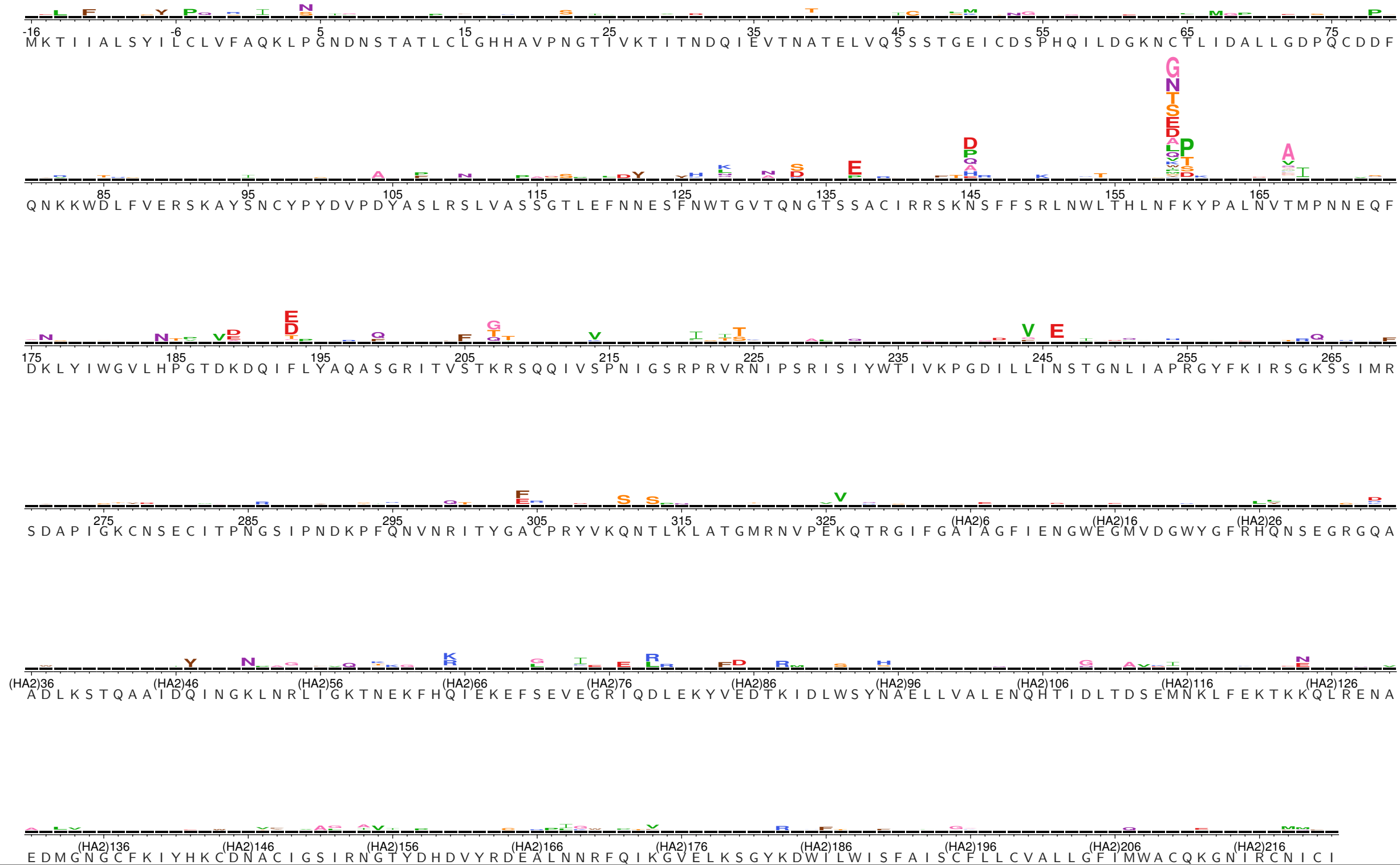

Supplement: Supplementary file 8. — The main figures in this paper just zoom in on the key sites of selection. These PDFs are also available at https://github.com/jbloomlab/map_flu_serum_Perth2009_H3_HA/tree/master/results/avgdiffsel/full_logo_plots. [file elife-49324-supp8.zip › Supplementary_file_8/antibody-3C06_diffsel.pdf]

differential selection = 4.5

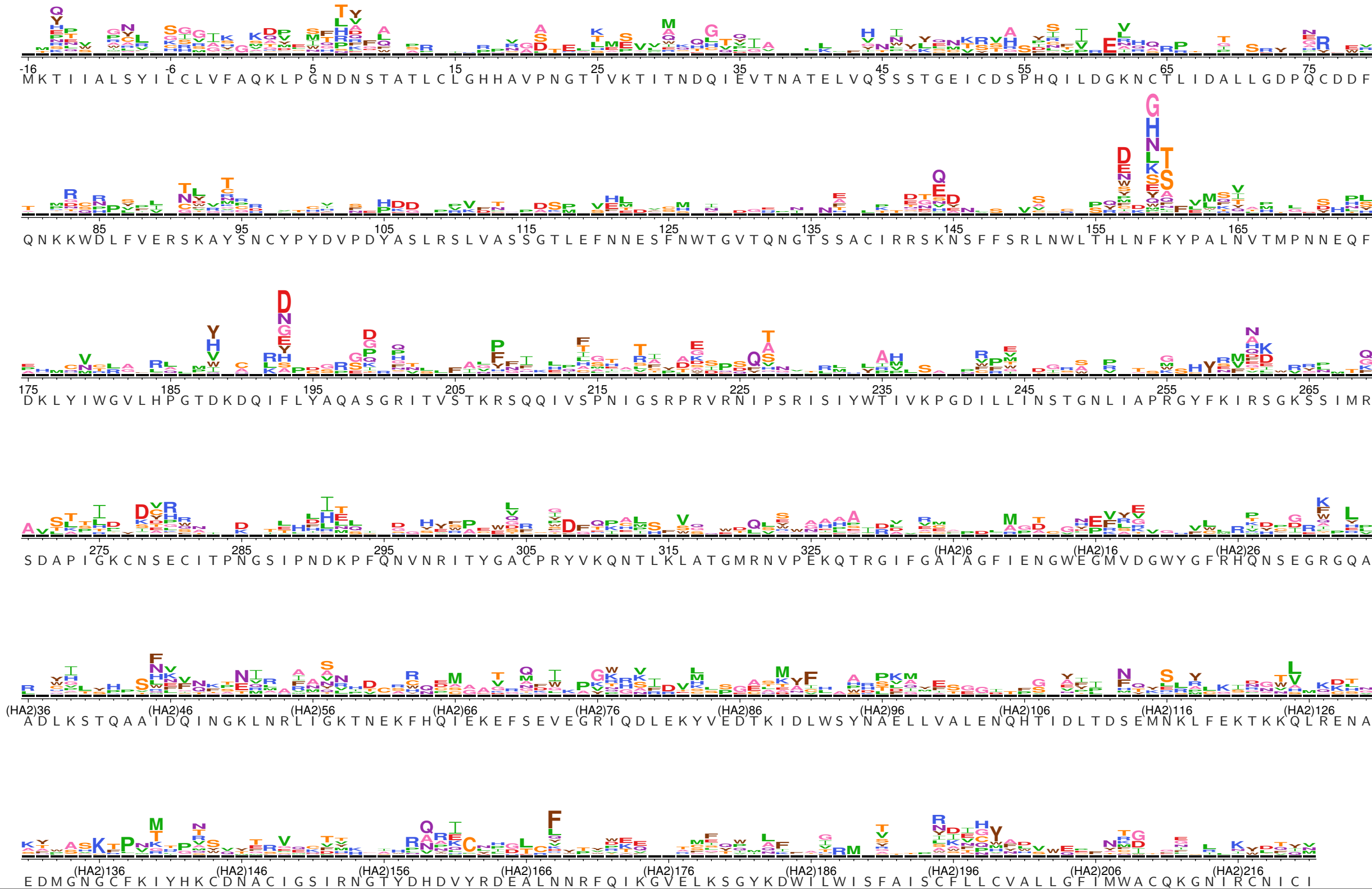

Supplement: Supplementary file 8. — The main figures in this paper just zoom in on the key sites of selection. These PDFs are also available at https://github.com/jbloomlab/map_flu_serum_Perth2009_H3_HA/tree/master/results/avgdiffsel/full_logo_plots. [file elife-49324-supp8.zip › Supplementary_file_8/2015-age-29-prevacc_diffsel.pdf]

differential selection = 1.9

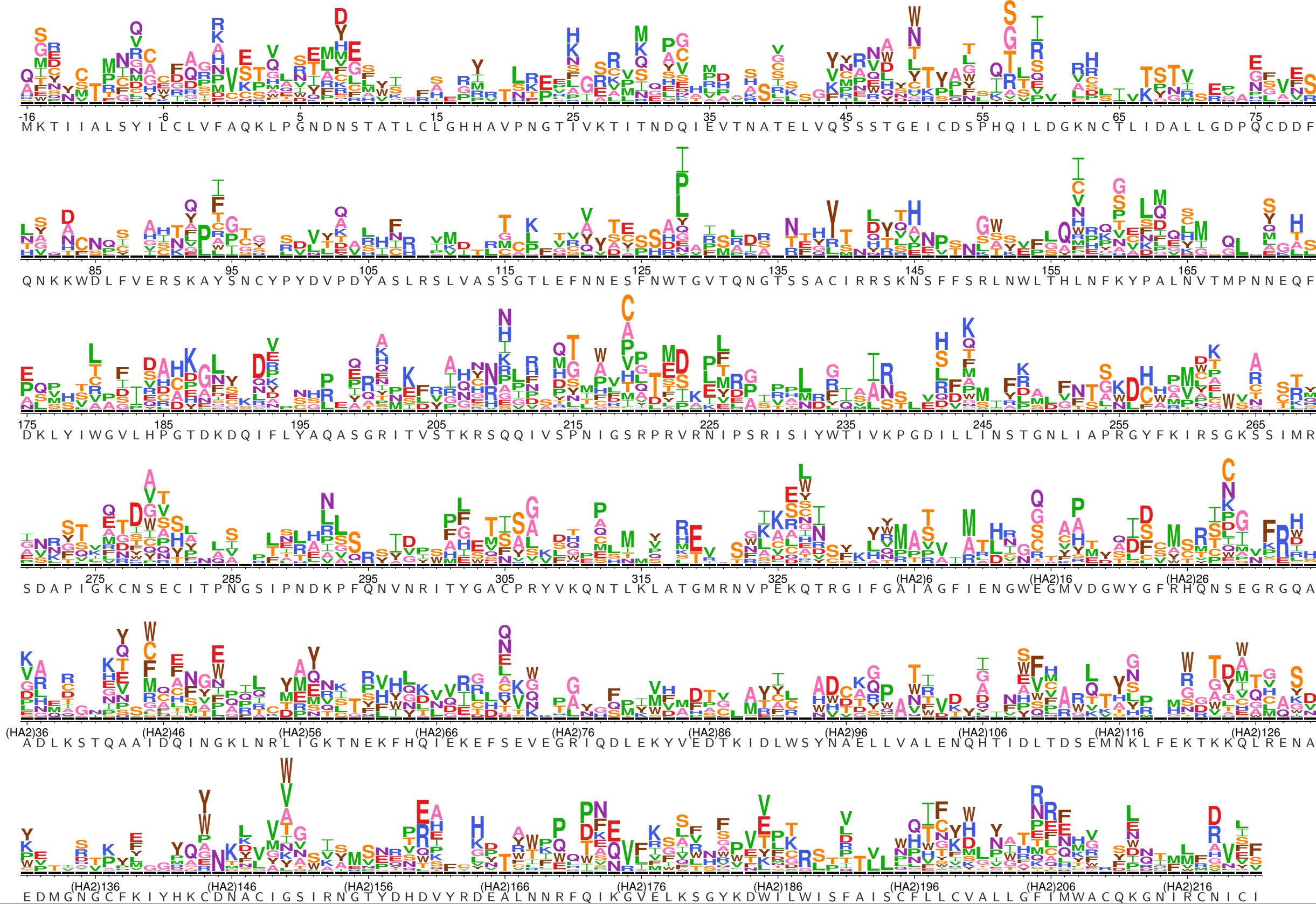

Supplement: Supplementary file 8. — The main figures in this paper just zoom in on the key sites of selection. These PDFs are also available at https://github.com/jbloomlab/map_flu_serum_Perth2009_H3_HA/tree/master/results/avgdiffsel/full_logo_plots. [file elife-49324-supp8.zip › Supplementary_file_8/ferret-Pitt-2-preinf_diffsel.pdf]

differential selection = 6.8

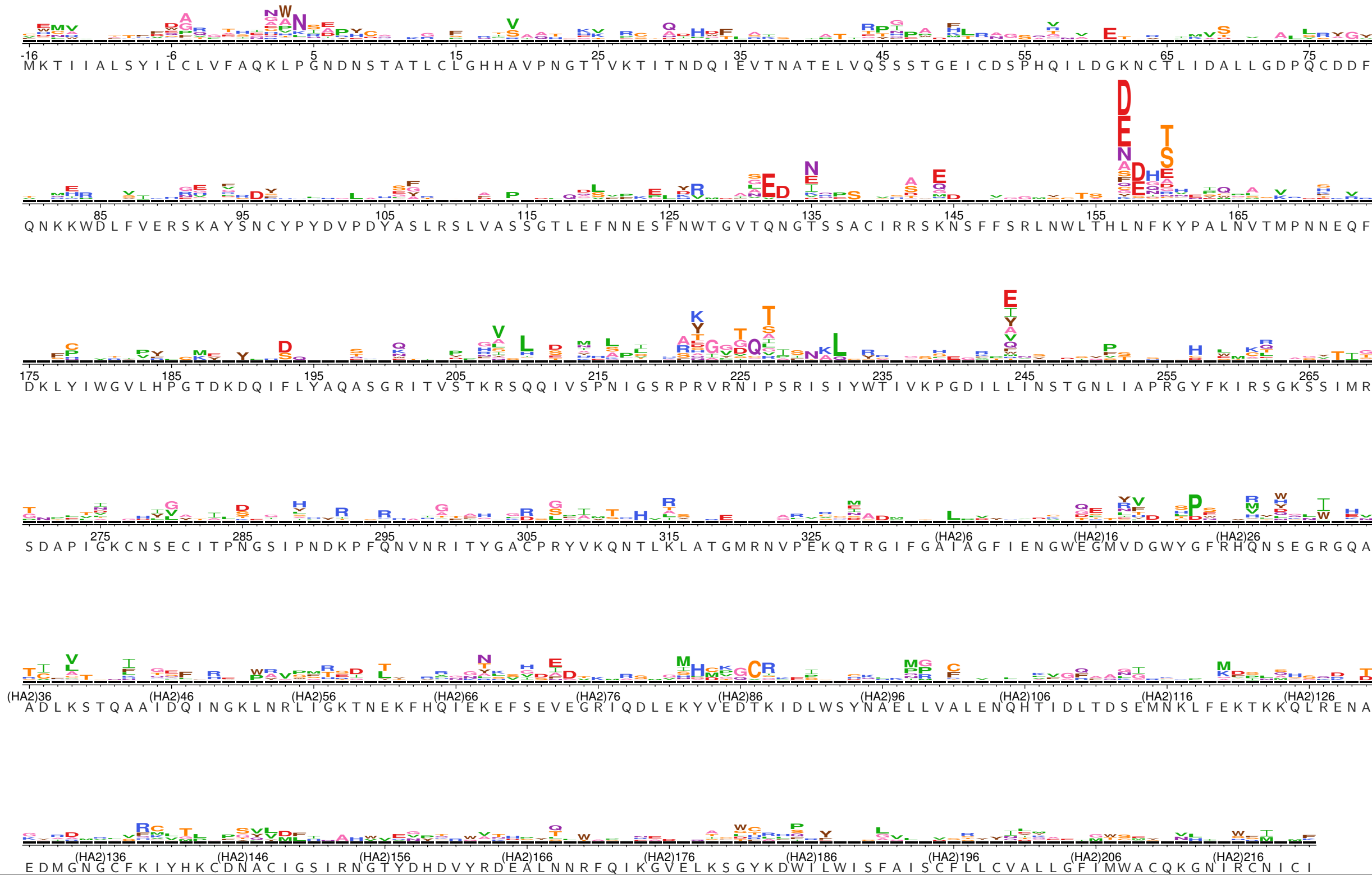

Supplement: Supplementary file 8. — The main figures in this paper just zoom in on the key sites of selection. These PDFs are also available at https://github.com/jbloomlab/map_flu_serum_Perth2009_H3_HA/tree/master/results/avgdiffsel/full_logo_plots. [file elife-49324-supp8.zip › Supplementary_file_8/2009-age-53-plus-2-months_diffsel.pdf]
