# Supplementary material for: Mapping person-to-person variation in viral mutations that escape polyclonal serum targeting influenza hemagglutinin
Source: eLife. 2019 Aug 27;8:e49324. doi: 10.7554/eLife.49324 (PMC6711711; doi:10.7554/eLife.49324)
Supplement: Supplementary file 8. — The main figures in this paper just zoom in on the key sites of selection. These PDFs are also available at https://github.com/jbloomlab/map_flu_serum_Perth2009_H3_HA/tree/master/results/avgdiffsel/full_logo_plots. [file 2015-age-25-vacc_diffsel.pdf › Supplementary_file_8/2015-age-25-vacc_diffsel.pdf]

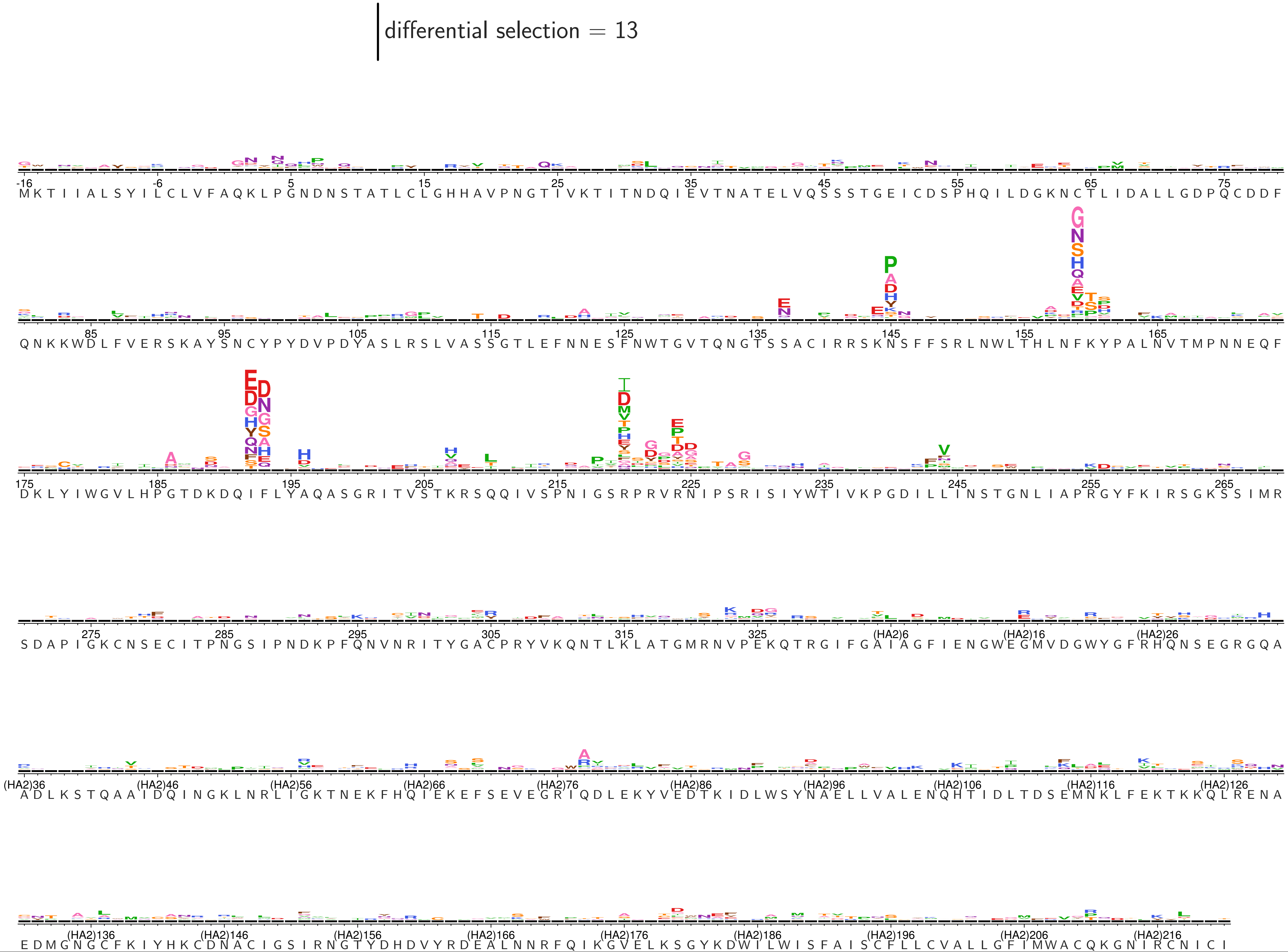
differential selection = 13
-16 -6 5 15 25 35 45 55 65 75
M K T I I A L S Y I L C L V F A Q K L P G N D N S T A T L C L G H H A V P N G T I V K T I T N D Q I E V T N A T E L V Q S S S T G E I C D S P H Q I L D G K N C T L I D A L L G D P Q C D D F
85 95 105 115 125 135 145 155 165
Q N K K W D L F V E R S K A Y S N C Y P Y D V P D Y A S L R S L V A S S G T L E F N N E S F N W T G V T Q N G T S S A C I R R S K N S F F S R L N W L T H L N F K Y P A L N V T M P N N E Q F
175 185 195 205 215 225 235 245 255 265
D K L Y I W G V L H P G T D K D Q I F L Y A Q A S G R I T V S T K R S Q Q I V S P N I G S R P R V R N I P S R I S I Y W T I V K P G D I L L I N S T G N L I A P R G Y F K I R S G K S S I M R
275 285 295 305 315 325 (HA2)6 (HA2)16 (HA2)26
S D A P I G K C N S E C I T P N G S I P N D K P F Q N V N R I T Y G A C P R Y V K Q N T L K L A T G M R N V P E K Q T R G I F G A I A G F I E N G W E G M V D G W Y G F R H Q N S E G R G Q A
(HA2)36 (HA2)46 (HA2)56 (HA2)66 (HA2)76 (HA2)86 (HA2)96 (HA2)106 (HA2)116 (HA2)126
A D L K S T Q A A I D Q I N G K L N R L I G K T N E K F H Q I E K E F S E V E G R I Q D L E K Y V E D T K I D L W S Y N A E L L V A L E N Q H T I D L T D S E M N K L F E K T K K Q L R E N A
(HA2)136 (HA2)146 (HA2)156 (HA2)166 (HA2)176 (HA2)186 (HA2)196 (HA2)206 (HA2)216
E D M G N G C F K I Y H K C D N A C I G S I R N G T Y D H D V Y R D E A L N N R F Q I K G V E L K S G Y K D W I L W I S F A I S C F L L C V A L L G F I M W A C Q K G N I R C N I C I
